# The RcDREB1E‐RcMYB28 module improves drought tolerance in *Rosa chinensis* via *RcLAR* / *RcCORA1* ‐mediated proanthocyanidin biosynthesis and stress responses

**DOI:** 10.1111/tpj.71169

**Published:** 2026-10-14

**Authors:** Yingjie Zhou, Linmei Chen, Huanyu Zhang, Yeni Chen, Jingjing Xin, Wen Chen, Yongyi Cui, Yuxiao Shen, Ping Luo

**Affiliations:** ^1^ College of Horticulture, Zhejiang A & F University Hangzhou China; ^2^ Key Laboratory of Quality and Safety Control for Subtropical Fruit and Vegetable Ministry of Agriculture and Rural Affairs, Zhejiang A & F University Hangzhou China; ^3^ Collaborative Innovation Center for Efficient and Green Production of Agriculture in Mountainous Areas of Zhejiang Province Zhejiang A & F University Hangzhou China; ^4^ College of Landscape Architecture, Henan Agricultural University Zhengzhou China

**Keywords:** *Rosa chinensis*, *RcMYB28*, *RcDREB1E*, proanthocyanidins, drought tolerance

## Abstract

Drought is widely recognized as one of the most severe abiotic stresses limiting plant growth and productivity. Proanthocyanidins (PAs), as key functional secondary metabolites, are central to enhancing plant tolerance to abiotic stresses. Nevertheless, the specific mechanisms underlying PA‐mediated drought resistance in rose remain largely unclear. Herein, we demonstrate that drought‐induced PA accumulation constitutes a conserved evolutionary mechanism across the plant kingdom, ranging from unicellular algae to angiosperms. Specifically, according to the drought‐responsive transcriptome dataset of *Rosa chinensis* ‘Old Blush’, an R2R3‐MYB transcription factor, RcMYB28, was identified. Overexpression of *RcMYB28* remarkably enhanced drought tolerance and induced a significant increase in PAs accumulation, whereas plants with silenced *RcMYB28* displayed the opposite phenotypic characteristics. Specifically, *RcMYB28* directly regulated the expression of leucoanthocyanidin reductase (*RcLAR*) and a cold and drought regulatory‐protein encoding gene (*RcCORA1*), thereby promoting PA synthesis and enhancing the stress response. Moreover, we identified RcDREB1E as a potential upstream transcriptional regulator of *RcMYB28*. RcDREB1E exerts a positive regulatory effect on drought tolerance and PAs accumulation via *RcMYB28* expression. Collectively, our results demonstrate a new transcriptional regulatory module (RcDREB1E‐RcMYB28) underlying drought‐triggered PAs accumulation, which offers valuable genetic resources for engineering enhanced drought tolerance in rose.

## Significance Statement

Drought seriously hampers plant performance, and proanthocyanidins (PAs) confer abiotic‑stress tolerance, yet the molecular mechanisms of PA‐mediated drought resistance are still poorly elucidated in rose. We identified RcMYB28 that directly activates *RcLAR* and *RcCORA1* to promote PA biosynthesis and drought tolerance, while upstream RcDREB1E functions through RcMYB28 to form the RcDREB1E‐RcMYB28 module for rose drought‐resistance improvement.

## INTRODUCTION

As a major abiotic stress, drought severely impairs plant growth and development, resulting in enormous economic losses in global agricultural and horticultural systems (Sun et al., 2018; Yuan et al., 2018). To counteract the detrimental impacts of drought, plants have evolved sophisticated multilayered regulatory mechanisms, encompassing the entire spectrum from drought stress signal perception to the orchestrated regulation of downstream physiological, biochemical, transcriptional, and metabolic adaptive responses (Yan et al., 2020). Targeted genetic engineering through the heterologous expression of key drought tolerance‐associated genes is widely recognized as a robust and effective strategy for breeding transgenic plant varieties with improved drought resistance (Zhang et al., 2018). In this regard, in‐depth elucidation of the physiological and molecular mechanisms regulating drought stress responses is essential for advancing both fundamental research on drought resistance and the targeted genetic improvement of horticultural crops.

Numerous studies have well documented that abiotic stresses also upregulate the expression of genes encoding metabolic enzymes, which, in turn, promotes the accumulation of a diverse array of secondary metabolites, including soluble sugars, proline, and polyamines (Dai et al., 2018; Khan et al., 2021). Proanthocyanidins (PAs) are secondary metabolites in the flavonoid biosynthesis pathway, with their biosynthesis relying on two key reductases: leucoanthocyanidin reductase (LAR) converts leucocyanidin to catechin, and anthocyanidin reductase (ANR) converts anthocyanidins to epicatechin (Passeri et al., 2017; Wang, Ran, et al., 2017; Wang, Xu, et al., 2017). Previous studies have shown that stress treatment upregulates the expression of *ANR* and *LAR*, and *ANR* or *LAR* overexpression resulted in enhanced drought and fungal resistance, implying that *ANR* and *LAR* are closely implicated in stress tolerance (Luo et al., 2016; Yuan et al., 2012). However, the mechanisms by which PAs exert feedback regulation on plant drought stress responses, as well as how transcription factors (TFs) involved in PAs regulation participate in mediating drought stress responses‐especially in horticultural crops such as rose remain largely unclear.

Growing evidence indicates that TFs are essential for stress response regulation, mediating the transduction of stress signals into cellular responses through orchestrated reprogramming of a target gene network (Li, Fu, et al., 2021; Li, Geng, et al., 2021; Song et al., 2024). MYB TFs possess a highly conserved N‐terminal DNA‐binding domain (BD) consisting of one to four imperfect repeats, each approximately 52 amino acids in length (An et al., 2018). MYB TFs are grouped into four subfamilies based on adjacent repeat number/arrangement: 1R‐MYB (MYB‐related), R2R3‐MYB (2R), R1R2R3‐MYB (3R), and 4R‐MYB (four tandem repeats, R1/R2 motifs) (Xu et al., 2015). Most plant *MYB* genes (encoding R2R3‐type MYB proteins) constitute the largest subgroup within the family, and these proteins are well established as pivotal mediators of defense responses to diverse abiotic stresses like drought, salinity, and extreme temperatures (Zhang, Jiang, et al., 2020; Zhang, Ye, et al., 2020). Members of the R2R3‐MYB subfamily in *Arabidopsis thaliana*, including AtMYB30, AtMYB60, and AtMYB96, are recognized for their involvement in conferring drought tolerance through the transcriptional regulation of multiple downstream genes (Dubos et al., 2010). Another investigation reported that TaMYB73, a MYB TF homolog from wheat (*Triticum aestivum*), confers increased salt tolerance when expressed in transgenic *A. thaliana*. This effect is likely mediated through the transcriptional upregulation of several stress‐inducible genes, such as *CBF3*, *RD29A*, and *RD29B* (He et al., 2012). Very recently, SlMYB13 of tomato was found to be a target by SlMYC2, the crucial and core TF in jasmonic acid signaling, and the resulting SlMYC2‐SlMYB13 complex plays a role in modulation of cold tolerance by directly upregulating the expression of *SlHSP17*.7 (Zhang et al., 2025). However, there is limited documentation of MYB TFs that regulate PA biosynthesis and are simultaneously induced under drought stress.

As one of the most popular ornamental species globally, *Rosa hybrida* holds significant economic importance due to its use in potted and cut‐flower forms across the hospitality, entertainment, and wellness sectors (Li, Fu, et al., 2021; Li, Geng, et al., 2021). As drought stress severely impairs rose growth and reduces its commercial value, investigating the drought‐resistance mechanisms of rose is urgently needed. The China rose, *Rosa chinensis* ‘Old Blush’, is a diploid cultivar that is easy to propagate. It is distinguished by its recurrent flowering habit, double‐petaled flowers, and a pleasant, lingering fragrance, making it an ideal subject for molecular biology and cross breeding studies (Raymond et al., 2018). Previous comprehensive transcriptomic profiling of ‘Old Blush’ under drought stress revealed that drought stress leads to significant changes in plant hormone metabolism, primary metabolism, and secondary metabolism—including the flavonoid biosynthesis pathway (the key pathway for PA synthesis) (Jia et al., 2021). Here, we present the functional identification and characterization of *RcMYB28*, isolated from the rose cultivar ‘Old Blush’, with respect to its role in drought tolerance. RcMYB28 directly interacted with the promoters of *RcLAR* and *RcCORA1*, leading to the activation of *RcLAR* and *RcCORA1* expression and subsequent PAs accumulation and osmotic adjustment, ultimately enhancing the tolerance of rose plants under drought stress. In addition, we showed that *RcMYB28* is a target gene of RcDREB1E, which plays a role in drought tolerance via PAs synthesis. Overall, the results of our study confirmed that the RcDREB1E‐RcMYB28 module regulates the response to drought stress through its effects on the PA biosynthesis.

## RESULTS

### Drought stress‐induced accumulation of PAs in rose

In *R. chinensis* ‘Old Blush’, a previous study identified extensive DEGs (BioProject ID: PRJNA722055) in response to drought stress (Jia et al., 2021). We also found that the secondary metabolism including phenylpropanoid metabolism and flavonoid biosynthesis was enriched at drought treatment for 60 days (Figure 1A). It should be noted that drought treatment upregulated the expression levels of *RcDFR*, *RcANR*, *RcLAR* and *RcANS* in PA biosynthesis of rose (Figure 1B). In addition, dehydration treatment also significantly induced *RhANR* and *RhLAR* expression in rose petals and leaves (Figure S1), correlating with elevated PA levels in these organs (Figure 1C,D). These data revealed that the drought stress induced the PAs accumulation in rose.

**Figure 1:**
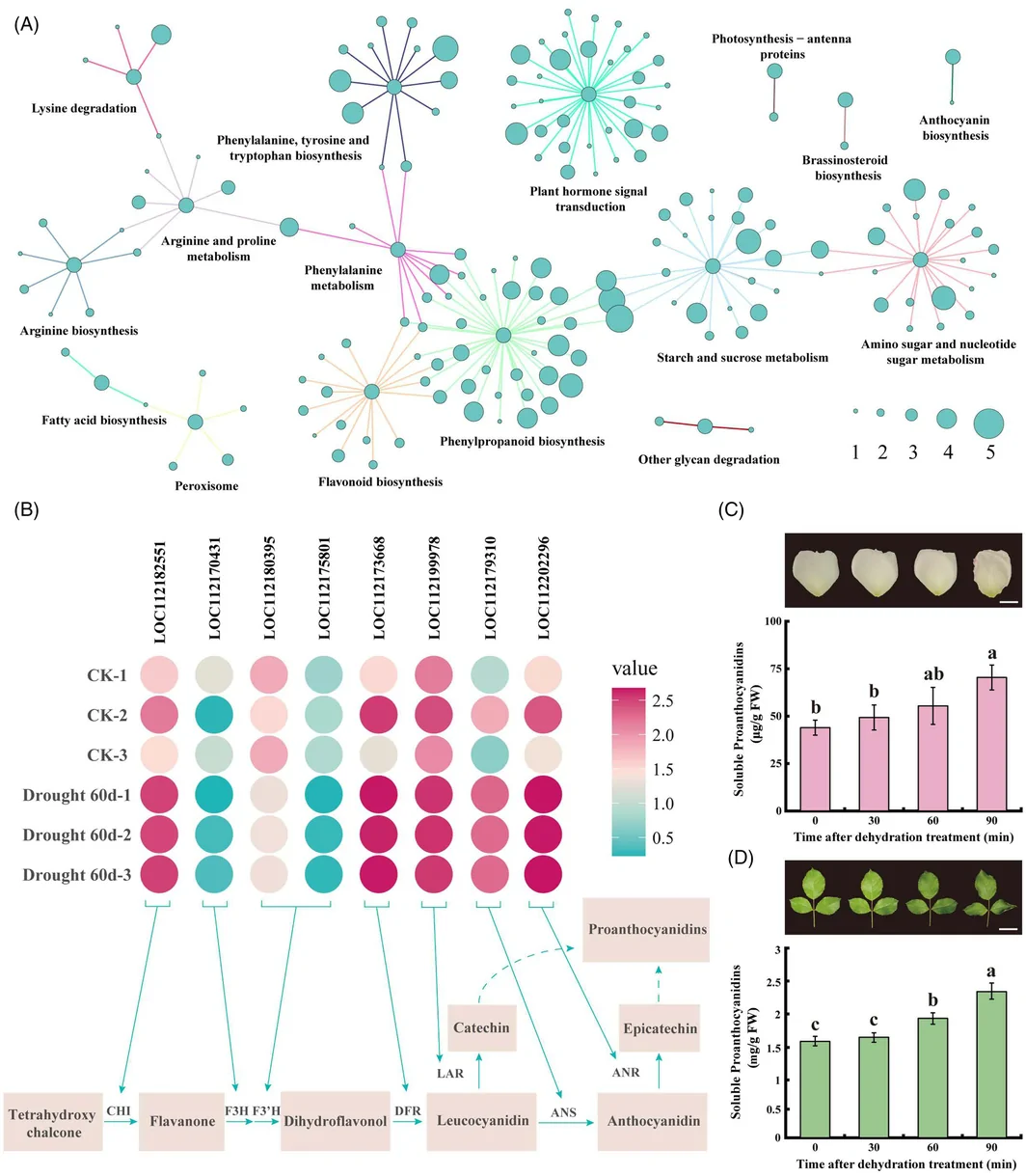
Differentially expressed genes (DEGs) of *Rosa chinensis* ‘Old blush’ under drought stress. (A) Significantly enriched gene pathways involving DEGs following the drought stress for 60 days. (B) Heatmap of proanthocyanidins (PAs) synthesis‐related DEGs in *R. chinensis* ‘Old blush’ under drought stress for 60 days. (C) The PAs levels of rose petals (*Rosa hybrida*) during the dehydration. (D) The PAs levels of rose leaves (*R. hybrida*) during the dehydration. Experiment was conducted using 3 biological replicates of 12 individual samples. Each of the samples had three technical replicates. Values are means ± SD from three independent biological replicates. Different letters indicated significant difference (*P* < 0.05) as obtained by one‐way anova and LSD test.

### Dehydration‐induced PAs accumulation is widely conserved

Given that PAs in rose were accumulated in response to dehydration, we further tested whether the accumulation of PAs in response to dehydration was a common mechanism conserved in species representing the major clades and subclades across the plant kingdom, namely *Plagiomnium acutum*, *Hypnum plumaeforme*, *Woodwardia japonica*, *Dryopteris erythrosora*, *Ginkgo biloba*, *Taxus baccata*, *Phalaenopsis amabilis*, *Hippeastrum vittatum*, *Dianthus caryophyllus*, *Magnolia denudata*, *Malva sylvestris*, *Oenothera speciosa*, *Hydrangea macrophylla*, *Oxalis articulata*, *Phlomis fruticosa*, *Argyranthemum frutescens*, *Rhododendron simii*, *Helianthus annuus*, *Impatiens hawkeri*, *Solanum lycopersicum*, *Petunia atkinsiana*, and *Glandularia tenera* (Figure 2A). Significant variations in PA content were observed among the harvested tissues of different plant species, with concentrations ranging from 4.3 to 2776.8 μg g^−1^ FW (Figure 2B). Specifically, *H. macrophylla* exhibited the highest PA content, while *P. fruticosa* showed the lowest (Figure 2B). Notably, the rates of PA accumulation also exhibited significant differences among different plant species. For example, those of *P. acutum*, *D. erythrosora*, *G. biloba*, *D. caryophyllus* and *P. fruticosa* reached the peak value at 60 min, while that of other species peaked at 90 min (Figure 2B). These results suggested the dehydration‐induced PAs accumulation is widely conserved in the plant kingdom.

**Figure 2:**
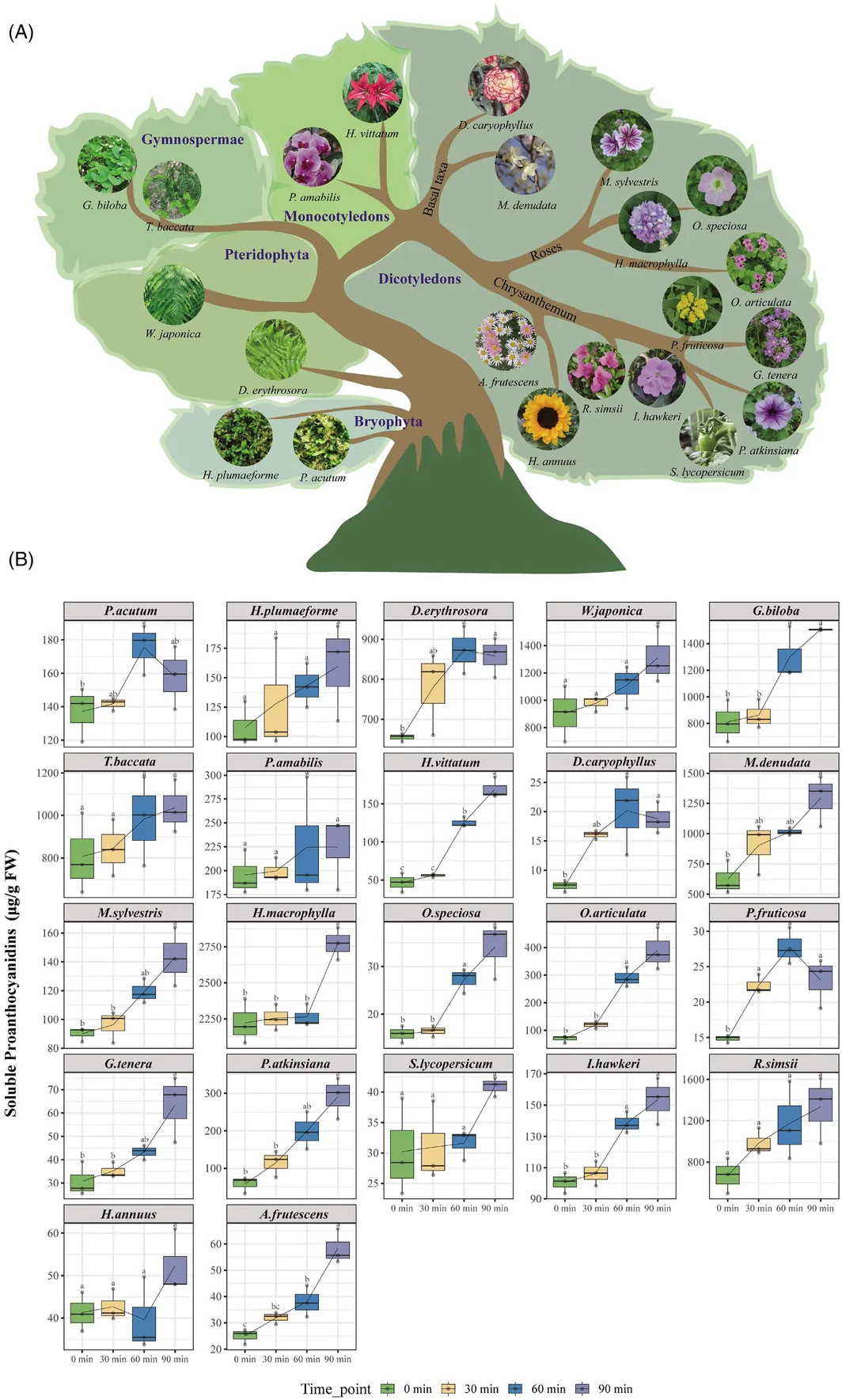
Proanthocyanidins (PAs) accumulation was induced by dehydration stress. (A) The species being studied represent the major clades and subclades in the plant kingdom. (B) PAs concentrations increased in most plants after exposure to dehydration. The experiment was conducted using 3 biological replicates of 12 individual samples. Each of the samples had three technical replicates. Values are means ± SD from three independent biological replicates. Different letters indicated significant difference (*P* < 0.05) as obtained by one‐way anova and LSD test.

### Molecular characterization of *RcMYB28*

As documented in previous work, MYB TFs serve as core regulators of PA biosynthesis, as typified by RrMYB5, RrMYB10, RcMYBPA2 and MdMYB23 (Li et al., 2019; Shen et al., 2019). We therefore investigated whether the upregulation of PA biosynthetic genes under drought stress is transcriptionally activated by MYB TFs in *R. chinensis*. Transcriptomic analysis revealed that over 2000 genes were differentially expressed, and 18 MYB family genes were significantly transcriptionally upregulated after 60 days of drought treatment (Figure S2). Phylogenetic analysis of the 18 *R. chinensis* MYB TFs revealed that LOC112171864 and LOC112185427 fell into the evolutionary branch of PA‐activating MYB TFs (Figure S3). Considering that the upregulation fold change of LOC112171864 was significantly higher than that of LOC112185427 under both 30‐ and 60‐day drought treatments (Figure S2), we therefore selected LOC112171864 (hereafter designated RcMYB28) as the core candidate gene for subsequent functional validation of its role in drought stress response.

The full‐length sequence of *RcMYB28* was amplified from the leaf cDNA of *R. chinensis* ‘Old Blush’, and subsequent sequence alignment and analysis revealed that this gene has a 921‐bp open reading frame (ORF), encoding a 306‐amino acid polypeptide (GenBank accession no: PZ262361). Multiple sequence alignment showed that RcMYB28 harbors a conserved R2R3 domain in the N‐terminal region, while the C‐terminal region is highly divergent among different MYB proteins (Figure S4).

To investigate the tissue‐specific expression pattern of *RcMYB28*, quantitative reverse transcriptase‐polymerase chain reaction (qRT‐PCR) was performed to measure its transcript abundance across multiple plant organs, specifically roots, stems, leaves, flowers, and fruits. The transcriptional levels of *RcMYB28* in flowers and leaves were higher than that in root, stem and fruits (Figure 3A). In addition, the transcript abundance of *RcMYB28* started to accumulate at 0.5 h post‐dehydration, and continued to increase steadily until the end of the treatment, with the final level being more than 6‐fold higher than the initial level (Figure 3B). The steady‐state mRNA level of *RcMYB28* began to accumulate within 1 h following NaCl treatment and exhibited a sustained upward trend over time (Figure 3C).

**Figure 3:**
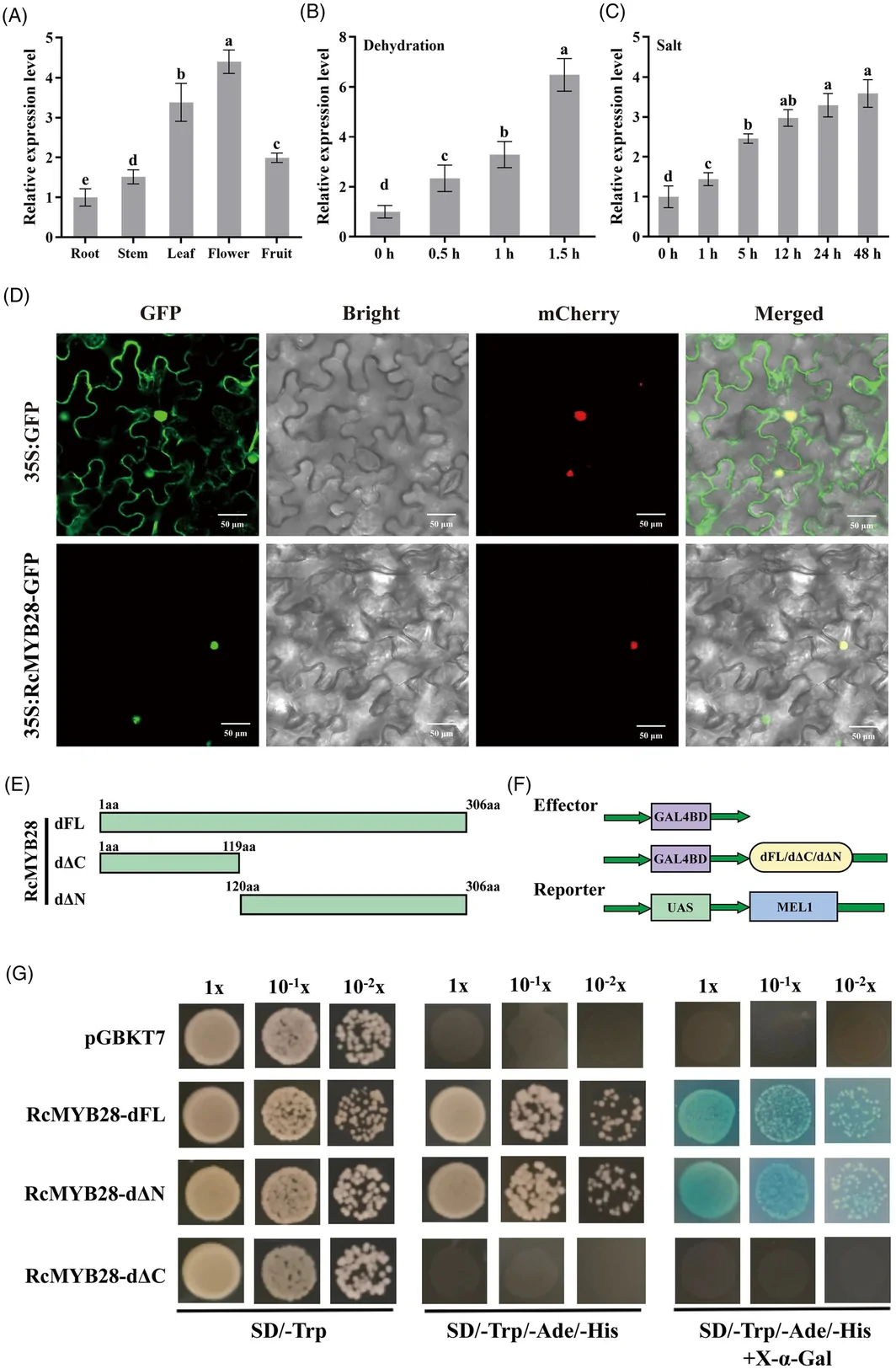
Expression pattern, subcellular localization and transcriptional activity of RcMYB28. (A) Transcript levels of *RcMYB28* in different rose tissues (roots, stems, leaves, flowers, and fruits). (B) Expression patterns of *RcMYB28* in response to dehydration. (C) Expression patterns of *RcMYB28* in response to salt. (D) RcMYB28 is localized in the nucleus, based on visualization of green fluorescent protein (GFP) in tobacco (*Nicotiana benthamiana*) leaves transformed with a fusion construct (35S:RcMYB28‐GFP) or empty vector (35S:GFP). Microscopic images were taken under bright field and fluorescence, VirD2NLS‐mCherry as a nuclear marker for co‐localization. The overlapped images are shown on the right. Bar = 50 μm. (E) Schematic diagrams of the full length (FL) and two truncated fragments (dΔN and dΔC) of RcMYB28 used for constructing vectors. (F) Effectors and reporter used for transcriptional activation activity assay. The FL and the two fragments (dΔN and dΔC) of RcMYB28 were introduced into the pGBKT7 vector, generating four effectors (pGBKT7‐FL/dΔN/dΔC). (G) Growth of yeast cells co‐transformed with different vectors on the SD/‐Trp, SD/‐Trp/‐Ade/‐His, and SD/‐Trp/‐Ade/‐His added with X‐α‐Gal. pGBKT7 vector was used as a negative control. 1×, 10^−1^× and 10^−2^× represent yeasts with original concentration (OD_600_ = 0.5), 10 and 100 folds of dilution, respectively. Values are means ± SD from three independent biological replicates. *RcGAPDH* was used as the internal control for qRT‐PCR analysis. Different letters indicated significant difference (*P* < 0.05) as obtained by one‐way anova and LSD test.

To investigate the subcellular distribution of RcMYB28, a GFP‐tagged construct encoding the full‐length *RcMYB28* coding sequence in‐frame with GFP was generated and used for localization analysis. Microscopic observations demonstrated that the RcMYB28‐GFP fusion protein emitted a strong green fluorescent signal, which was localized solely in the nucleus; in contrast, the GFP protein alone showed a uniform fluorescent distribution across the entire cell (Figure 3D). Thus, the RcMYB28 protein was localized to the nucleus.

To assess the transcriptional regulatory potential of RcMYB28, a yeast transactivation assay was conducted, testing its ability to activate reporter gene expression *in vivo*. Full‐length (dFL) and truncated fragments (dΔC, dΔN) of RcMYB28 were, respectively, ligated to the downstream of the GAL4 BD in the pGBKT7 vector, followed by transformation into the yeast AH109 strain (Figure 3E,F). All yeast cells exhibited normal growth on SD/‐Trp medium (Figure 3G). In contrast, only the yeast cells co‐transformed with the full‐length RcMYB28 (RcMYB28‐FL) and the N‐terminal truncated RcMYB28 (RcMYB28‐dΔN) were able to survive on the SD/‐Trp/‐Ade/‐His medium (Figure 3G). These findings revealed that RcMYB28 opposed transactivation activity and the transactivation domain was situated at the C‐terminal region of the protein.

### Overexpression of *RcMYB28* induces PAs accumulation in tobacco

To further confirm the role of *RcMYB28* in the process of PA biosynthesis, we introduced 35S::*RcMYB28* into tobacco. Two independent *RcMYB28* overexpression lines (OE3 and OE5), exhibiting the highest transcript levels of *RcMYB28*, were selected for subsequent analyses (Figure 4A). Compared with the red petals of WT, the petals of *RcMYB28*‐overexpressing plants displayed a white or light pink color (Figure 4B). However, leaf phenotypes showed no noticeable differences between transgenic and wild‐type (WT) plants (Figure S5). Significantly elevated PAs contents were observed in petals and leaves of *RcMYB28*‐overexpressing lines relative to the WT (Figure 4C,D). Expression analysis showed that the key genes encoding PA biosynthetic enzymes, *NtANR* and *NtLAR*, exhibited an increasing expression trend in both petals and leaves of *RcMYB28*‐overexpressing tobacco (*Nicotiana tabacum*) (Figure 4E,F). For anthocyanin biosynthesis‐related genes, *NtDFR* and *NtANS* were upregulated in petals of the transgenic lines, while *NtUFGT* was downregulated (Figure 4E; Figure S6). No significant difference was observed in the expression levels of *NtDFR*, *NtANS*, or *NtUFGT* in leaves compared with WT controls (Figure 4F; Figure S6). Collectively, these data suggest that ectopic expression of *RcMYB28* in tobacco could partially induce flavonoid pathway gene expression and enhance PAs accumulation.

**Figure 4:**
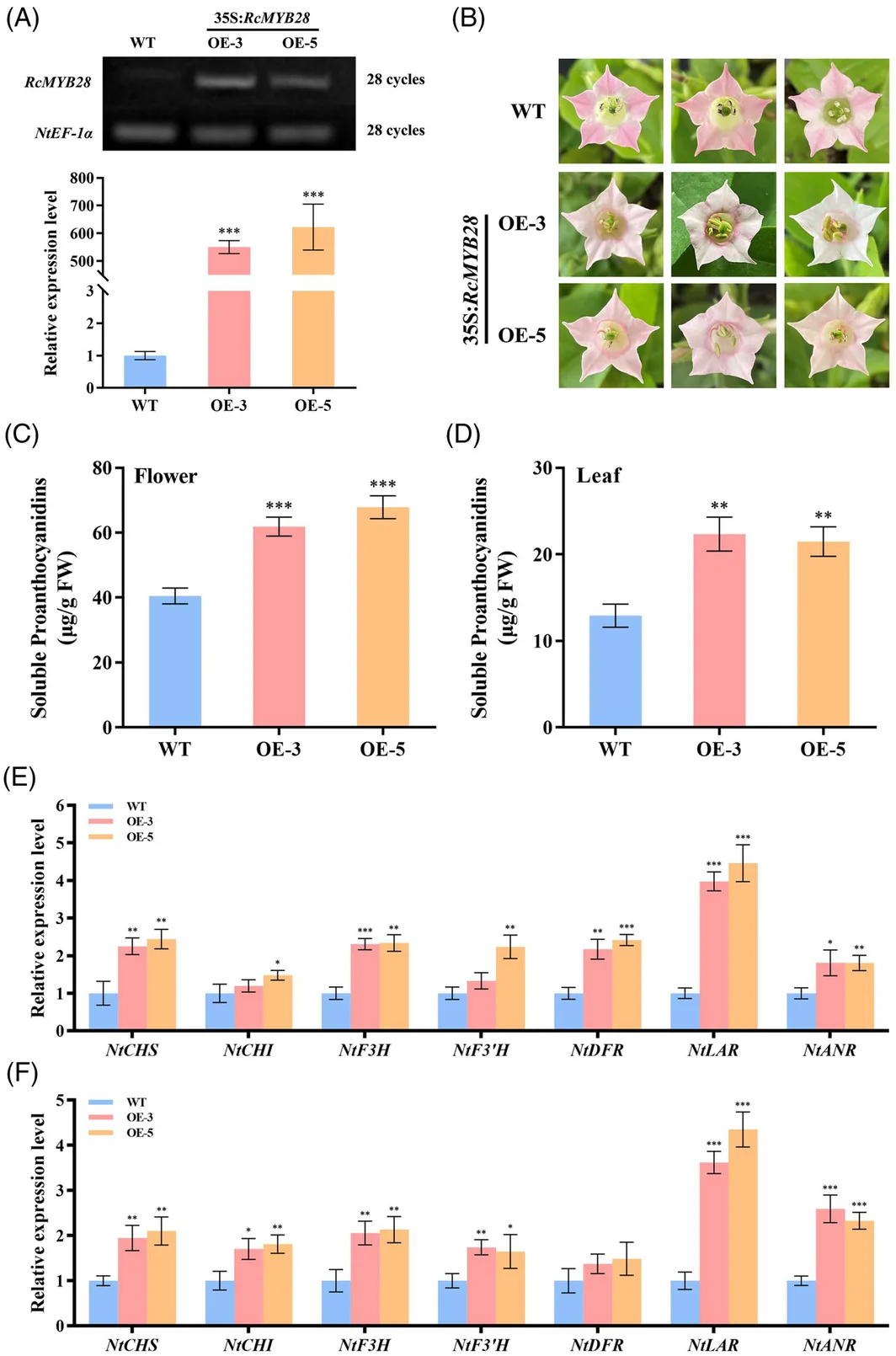
Functional characterization of *RcMYB28* overexpression in tobacco. (A) Semi‐quantitative RT‐PCR and qRT‐PCR analysis of *RcMYB28* transcript level between *RcMYB28* overexpression tobacco lines and wild‐type (WT). (B) Pigmentation of flower petals of WT and *RcMYB28*‐transgenic tobacco plants. (C) Proanthocyanidin (PA) levels in the flowers of *RcMYB28*‐transgenic tobacco. (D) PA levels in the leaves of *RcMYB28*‐transgenic tobacco. (E) qRT‐PCR analysis of flavonoid‐related gene expression in the flowers of *RcMYB28*‐transgenic tobacco. (F) qRT‐PCR analysis of flavonoid‐related gene expression in the leaves of *RcMYB28*‐transgenic tobacco. Experiment was conducted using 3 biological replicates of 15 individual samples. Each of the samples had three technical replicates. Values are means ± SD from three independent biological replicates. Statistical significance was determined using a Student's *t*‐test (*P < 0.05, **P < 0.01, ***P < 0.001).

### Overexpression of *RcMYB28* enhances drought tolerance in transgenic tobacco

Given that drought stress triggered the most significant induction of *RcMYB28* transcription, we speculated that *RcMYB28* may exert a role in the plant's response to drought stress. First, 20‐day‐old *in vitro* seedlings were exposed to dehydration in an ambient condition. The leaves of WT exhibited more severe wilting than those of the transgenic lines (Figure 5A). Despite a continuous reduction in fresh weight (FW) across all genotypes, the transgenic lines exhibited a markedly lower rate of water loss compared with the WT (Figure 5B). As illustrated in Figure 5C, transgenic lines exhibited significantly lower malondialdehyde (MDA) accumulation relative to the WT. Electrolyte leakage (EL), a key marker of membrane damage, was significantly higher in WT (41.2%) than in OE‐3 (28.0%) and OE‐5 (29.1%), suggesting more severe membrane injury in WT (Figure 5D). Collectively, these results indicated that the transgenic lines exhibited enhanced tolerance to dehydration.

**Figure 5:**
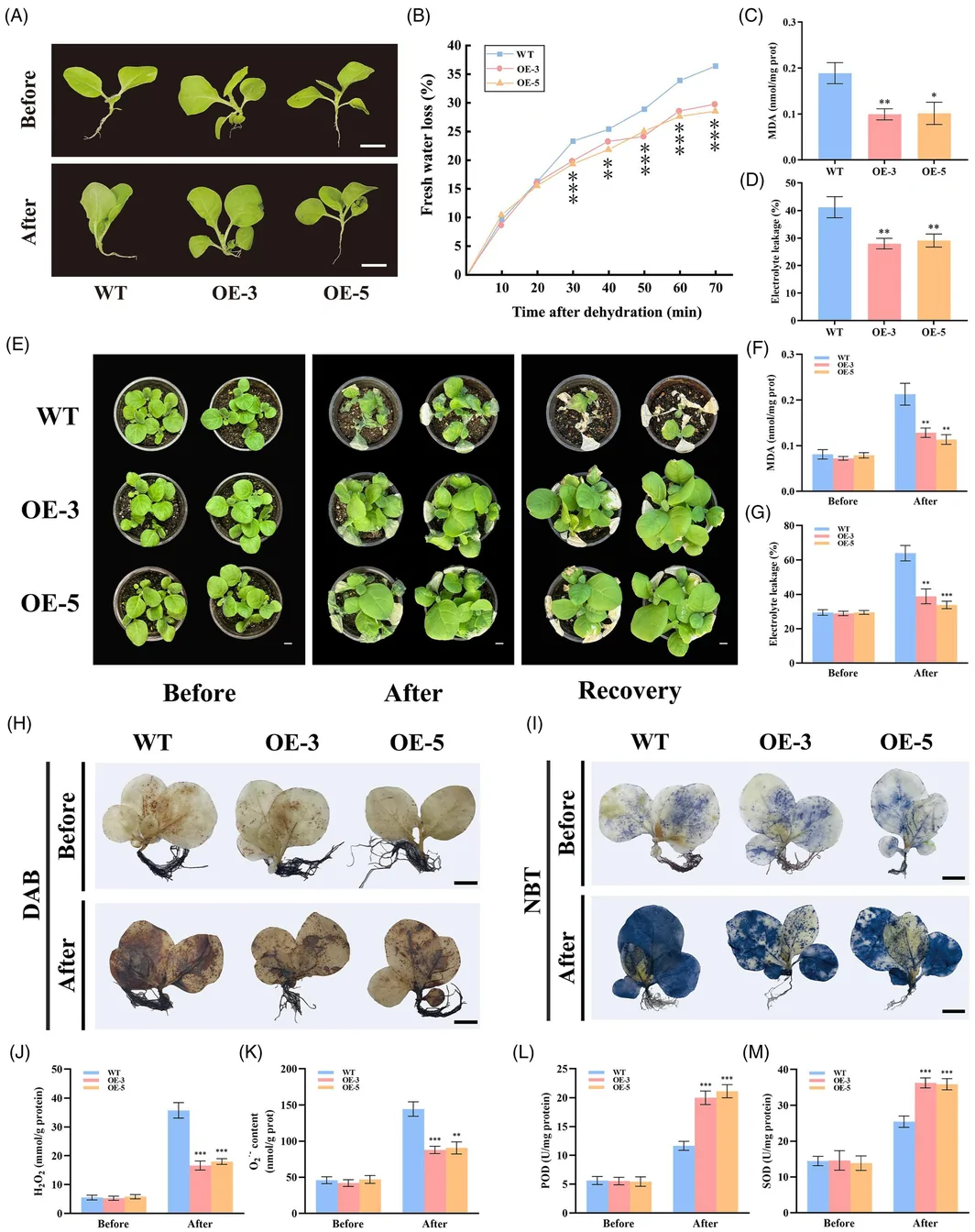
Overexpression of *RcMYB28* confers enhanced drought tolerance in transgenic tobacco plants. (A) Representative images of wild‐type (WT), OE‐3, and OE‐5 after dehydration for 70 min. (B) Time‐course fresh water loss of WT, OE‐3, and OE‐5 during 70‐min dehydration. (C, D) Malondialdehyde (MDA) contents (C) and electrolyte leakage (D) in the two lines measured after dehydration for 70 min. (E) The phenotype of tobacco transgenic (OE‐3 and OE‐5) and WT plants before and after drought stress. (F, G) MDA contents (F) and electrolyte leakage (G) in the two lines measured after drought. (H, I) Histochemistry was stained with nitrotetrazolium blue chloride (NBT) and 3,3′‐diaminobenzidine (DAB) to assess the accumulation of H_2_O_2_ (H) and O_2_^−^ (I), respectively, before and after drought stress. (J, K) Quantitative measurements of H_2_O_2_ (J) and O_2_^−^ (K) in transgenic lines and WT before and after drought stress. (L, M) Peroxidase (POD) (L) and superoxide dismutase (SOD) (M) activities of the transgenic lines and WT were determined before and after drought stress. The experiment was conducted using 3 biological replicates of 15 individual samples. Each of the samples had three technical replicates. Values are means ± SD from three independent biological replicates. Statistical significance was determined using a Student's *t*‐test (*P < 0.05, **P < 0.01, ***P < 0.001).

30 day‐old soil‐grown tobacco plants also were carried out to assess the tolerance of long‐term water stress (drought). Only a few WT tobacco plants survived following recovery, while the majority of transgenic plants recovered growth despite exhibiting some leaf damage (Figure 5E). Consistent with their enhanced drought tolerance, the RcMYB28‐overexpressing transgenic lines exhibited significantly lower EL and MDA contents than the WT following drought treatment (Figure 5F,G). Before exposure to drought stress, the transgenic lines and WT showed comparable levels of nitrotetrazolium blue chloride (NBT) and 3,3′‐diaminobenzidine (DAB) staining (Figure 5H,I). Upon exposure to drought stress, the transgenic lines exhibited significantly weaker staining than the WT, consistent with reduced reactive oxygen species (ROS) accumulation (Figure 5H,I). In agreement with the observed cold‐induced ROS staining patterns, biochemical quantification showed that both transgenic lines had markedly reduced O_2_^−^ and H_2_O_2_ contents relative to the WT (Figure 5J,K). As principal components of the antioxidant defense system, superoxide dismutase (SOD) and peroxidase (POD) are critical for sustaining intracellular ROS homeostasis. During drought treatment, transgenic lines exhibited markedly elevated SOD and POD activities compared with the WT (Figure 5L,M). Collectively, these results indicate that ectopic expression of *RcMYB28* in tobacco confers a marked improvement in tolerance to drought stress.

### Trans‐overexpression of *RcMYB28* results in dehydration tolerance in rose petals

To further verify the role of *RcMYB28* in dehydration tolerance, *RcMYB28* was also trans‐overexpressed in rose petals (Figure 6A). As shown in Figure 6B, the transgenic lines exhibited substantially stronger DMACA staining compared with the empty vector (EV) control. In accordance with the staining, quantitative determination demonstrated that petals of transgenic lines accumulated significantly higher levels of PAs compared with the EV (Figure 6C). As a result, the expression of *RhANR* and *RhLAR* in overexpressed petals was significantly enhanced compared to the EV (Figure 6D,E). Collectively, these results indicate that overexpression of *RcMYB28* positively regulates PA biosynthesis in rose petals.

**Figure 6:**
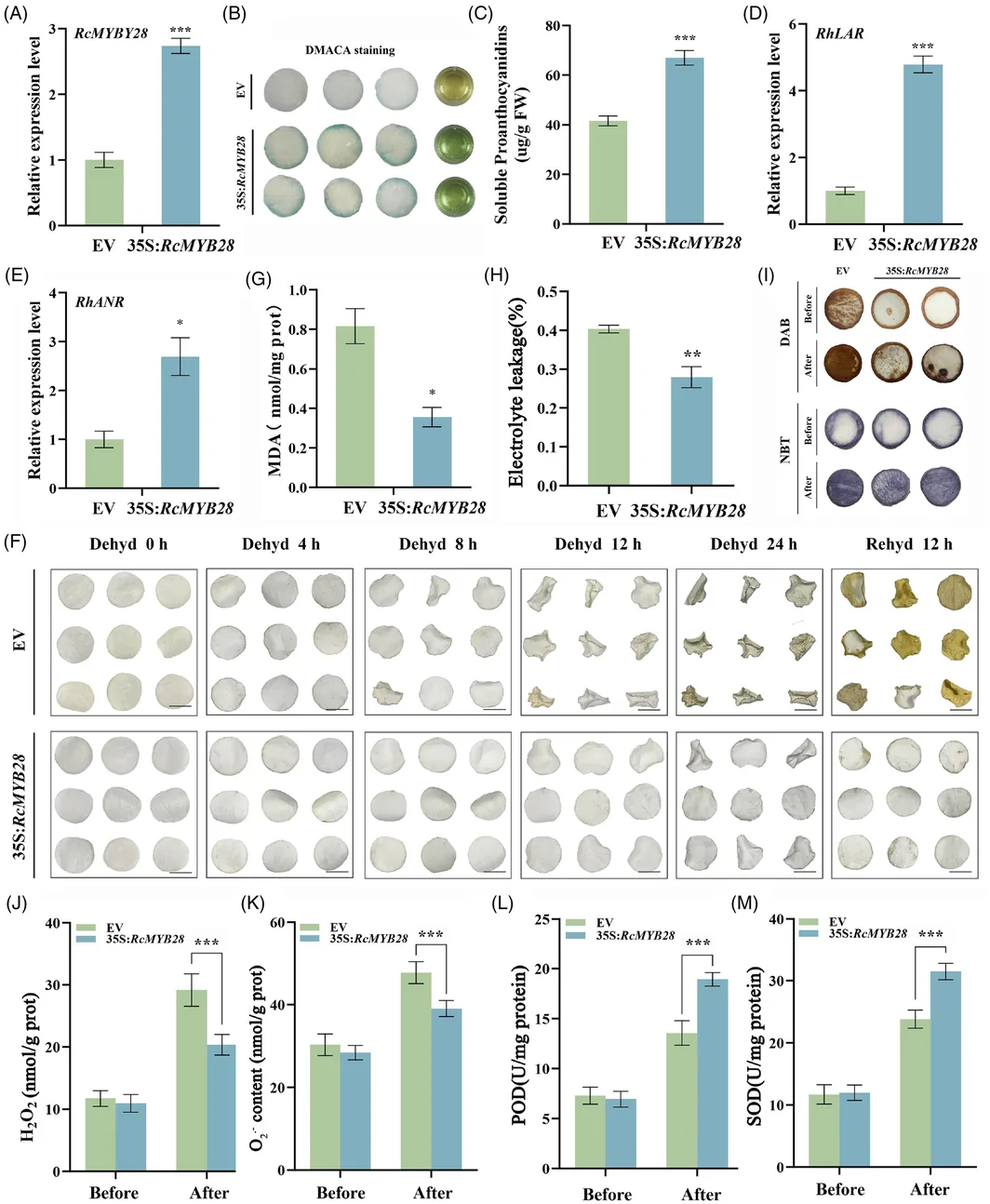
Dehydration tolerance assay of *RcMYB28‐*overexpressing rose petals. (A) Expression levels of *RcMYB28* in empty vector control (EV) and transgenic rose petals. (B) DMACA staining of transgenic rose petals. (C) Quantification of proanthocyanidin (PA) levels in transgenic rose petals. (D, E) The expression levels of *RhLAR* (D) and *RhANR* (E) in EV and *RcMYB28‐*overexpressing rose petals. (F) Phenotypes of *RcMYB28* transgenic rose petal discs (35S:*RcMYB28*) and EV control samples dehydrated for 0, 4, 8, 12, 24 h and followed by rehydration for 12 h. The rose petal discs were punched symmetrically in the middle of the petals using a 1.5‐cm hole punch. (G, H) Malondialdehyde (MDA) contents (G) and electrolyte leakage (H) in the *RcMYB28* transgenic rose petal discs measured after dehydration. (I) *In situ* accumulation of H_2_O_2_ and O_2_^−^ of EV and *RcMYB28* transgenic rose petals, as revealed by histochemical staining with 3,3′‐diaminobenzidine (DAB) and nitrotetrazolium blue chloride (NBT), before (upper panels) and after (lower panels) dehydration. (J, K) Quantitative measurements of H_2_O_2_ (J) and O_2_^−^ (K) in *RcMYB28* transgenic rose petals and EV before and after dehydration. (L, M) Peroxidase (POD) (L) and superoxide dismutase (SOD) (M) activities of the *RcMYB28* transgenic rose petals and EV were determined before and after dehydration. Twelve petal discs were taken as one biological replicate and three biological replicates were used for gene expression analysis and physiological experiment. Each of the samples had three technical replicates. Values are means ± SD from three independent biological replicates. Statistical significance was determined using a Student's *t*‐test (*P < 0.05, **P < 0.01, ***P < 0.001).

Following 24 h of dehydration, petal discs of the 35S:*RcMYB28* lines exhibited less shrinkage relative to EV control (Figure 6F). Moreover, the petals expressing 35S:*RcMYB28* had lower EL and MDA levels as compared to the EV (Figure 6G,H). Histochemical staining showed that the transgenic lines accumulated markedly lower levels of H_2_O_2_ and O_2_ compared with the EV (Figure 6I–K). Without the dehydration, the POD and SOD activities were similar to those of the EV (Figure 6L,M). However, under dehydration stress, the activities of both enzymes were significantly higher in the transgenic lines than in the EV (Figure 6L,M). These results indicated that overexpression of *RcMYB28* led to enhanced dehydration tolerance in the rose petals.

### Overexpression of *RcMYB28* results in drought tolerance in *Rosa rugosa*

*RcMYB28* was next overexpressed in somatic embryos of *R. rugosa* ‘Bao white’, and those (OE‐2 and OE‐4) with the highest transgene expression were subjected to additional phenotypic and molecular characterization (Figure 7A). In addition, RcMYB28 from *R. chinensis* ‘Old blush’ and RrMYB28 from *R. rugosa* ‘Bao white’ share 96.08% sequence similarity (Figure S7). PA quantification demonstrated that *RcMYB28*‐overexpressing embryos accumulated more PAs than WT (Figure 7B). Similar to the WT, the *RcMYB28*‐transgenic embryos displayed a light yellow color, with no obvious phenotypic difference between the two groups (Figure 7C). Upon DMACA staining, the transgenic embryos appeared deep blue, while the WT embryos were stained light blue (Figure 7C). qRT‐PCR analysis of PA biosynthetic genes showed that the expressions of *RrLAR* and *RrANR* in transgenic embryos were significantly increased (Figure 7D,E). These data revealed that *RcMYB28* overexpression induces PAs accumulation in somatic embryos of *R. rugosa*.

**Figure 7:**
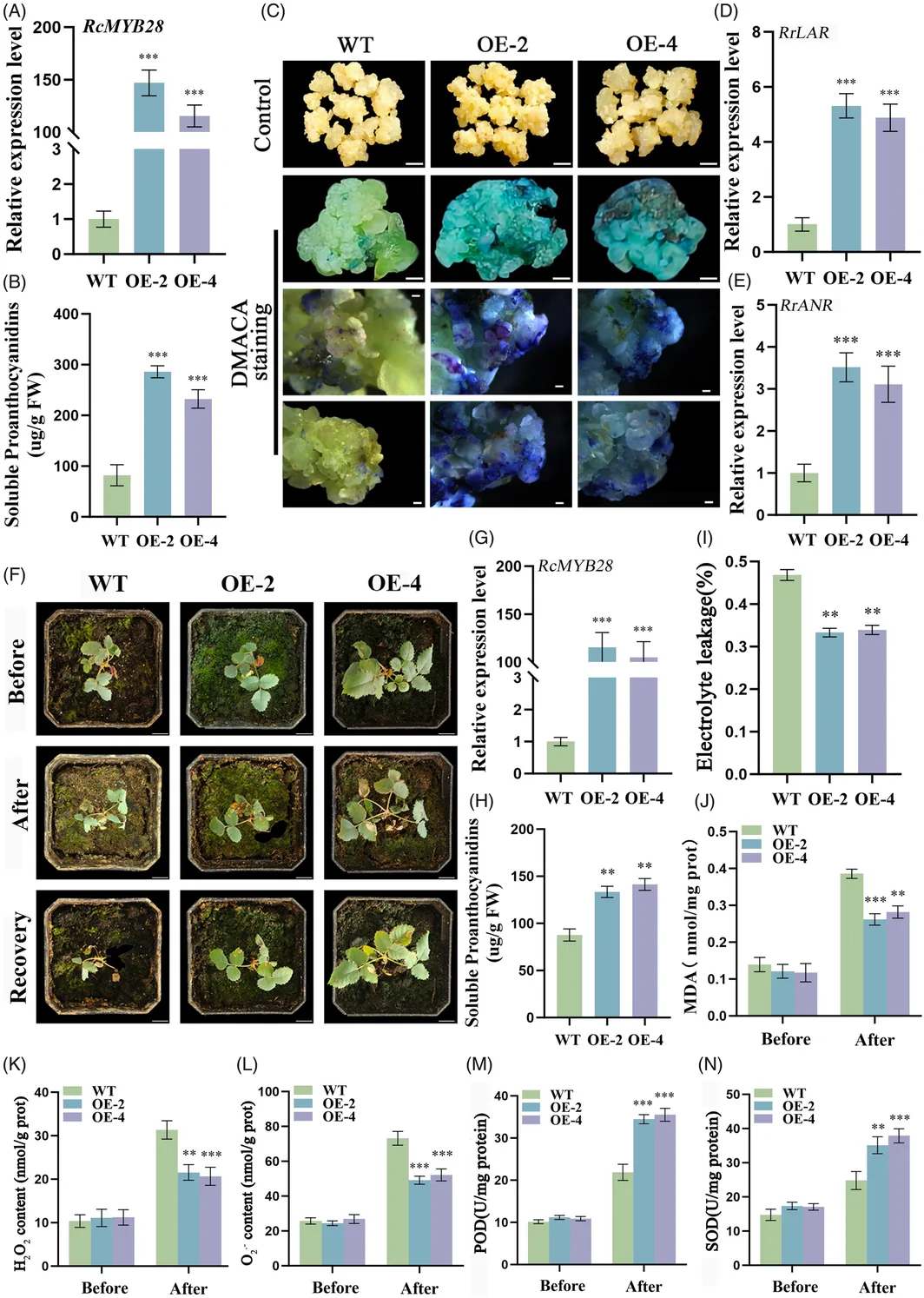
Overexpression of *RcMYB28* in *Rosa rugosa* ‘Bao white’ confers enhanced drought tolerance. (A) Expression levels of *RcMYB28* in wild‐type (WT) and transgenic rose somatic embryos (*R. rugosa* ‘Bao white’). (B) Proanthocyanidin (PA) levels in the somatic embryos of WT and transgenic lines. (C) DMACA‐stained somatic embryos of WT and transgenic lines. (D, E) The expression levels of *RrLAR* (D) and *RrANR* (E) in WT and *RcMYB28‐*overexpressing rose somatic embryos. (F) The phenotype of rose transgenic (OE‐2 and OE‐4) and WT plants before and after drought stress. (G) Expression levels of *RcMYB28* in WT and *RcMYB28‐*overexpressing rose lines (*R. rugosa* ‘Bao white’). (H) PA levels in the leaves of WT and transgenic lines. (I, J) Electrolyte leakage (I) and malondialdehyde (MDA) contents (J) in the two lines measured after drought. (K, L) Quantitative measurements of H_2_O_2_ (K) and O_2_^−^ (L) in transgenic lines and WT before and after drought stress. (M, N) Peroxidase (POD) (M) and superoxide dismutase (SOD) (N) activities of the transgenic lines and WT were determined before and after drought stress. Experiment was conducted using three biological replicates of five individual samples. Each of the samples had three technical replicates. Values are means ± SD from three independent biological replicates. Statistical significance was determined using a Student's *t*‐test (**P < 0.01, ***P < 0.001).

In addition, the *RcMYB28*‐overexpressing seedlings of rose (*R. rugosa* ‘Bao white’) generated from somatic embryos were used for drought tolerance assessment. After exposure to drought stress and subsequent recovery under ambient temperature conditions, the WT plants showed serious leaf wilting and drooping, whereas the OE lines remained viable, with few leaves showing wilting symptoms (Figure 7F,G). It should be noted that the PAs levels in the leaves of *RcMYB28*‐overexpressing rose plants were significantly higher than in WT (Figure 7H). In agreement with the observed phenotypes, drought‐treated transgenic plants exhibited markedly reduced EL and MDA contents relative to the WT (Figure 7I,J). Additionally, the ROS levels in the WT were significantly higher than in *RcMYB28*‐overexpressing lines after drought stress (Figure 7K,L). During drought treatment, transgenic lines exhibited markedly elevated POD and SOD activities compared with the WT (Figure 7M,N). Collectively, these findings demonstrate that *RcMYB28* overexpression leads to enhanced drought tolerance in roses.

### RcMYB28 directly regulates *RcLAR* and *RcCORA1*

qRT‐PCR analysis was conducted to evaluate the expression levels of six stress‐responsive genes in WT and *RcMYB28*‐overexpressing rose plants (*R. rugosa* ‘Bao white’) under both normal conditions and drought stress (Figure S8). It should be noted that the *CORA1* transcript level was prominently upregulated in *RcMYB28*‐overexpressing rose plants compared with the WT (Figure S8). In addition, the steady‐state mRNA levels of *ANR* and *LAR* genes in *RcMYB28*‐overexpressing rose plants were significantly higher than those in the WT plants. Taken together, we speculated that *ANR*, *LAR*, and *CORA1* genes might be the potential target genes that are regulated by *RcMYB28*. To explore whether RcMYB28 interacts with *RcANR*, *RcLAR*, and *RcCORA1* promoters, yeast one‐hybrid (Y1H) assay was conducted (Figure 8A). Yeast cells co‐transformed with RcMYB28‐AD and *proRcLAR*‐pHIS2.1 or *proRcCORA1*‐pHIS2.1 grew well on SD/‐His/‐Leu/‐Trp screening medium supplemented with 3‐AT (Figure 8B). However, yeast cells co‐transformed with RcMYB28‐AD and *proRcANR*‐pHIS2.1 did not grow under the same conditions (Figure 8B). These results revealed that RcMYB28 could bind to the *RcLAR* and *RcCORA1* promoters, but not the *RcANR* promoter.

**Figure 8:**
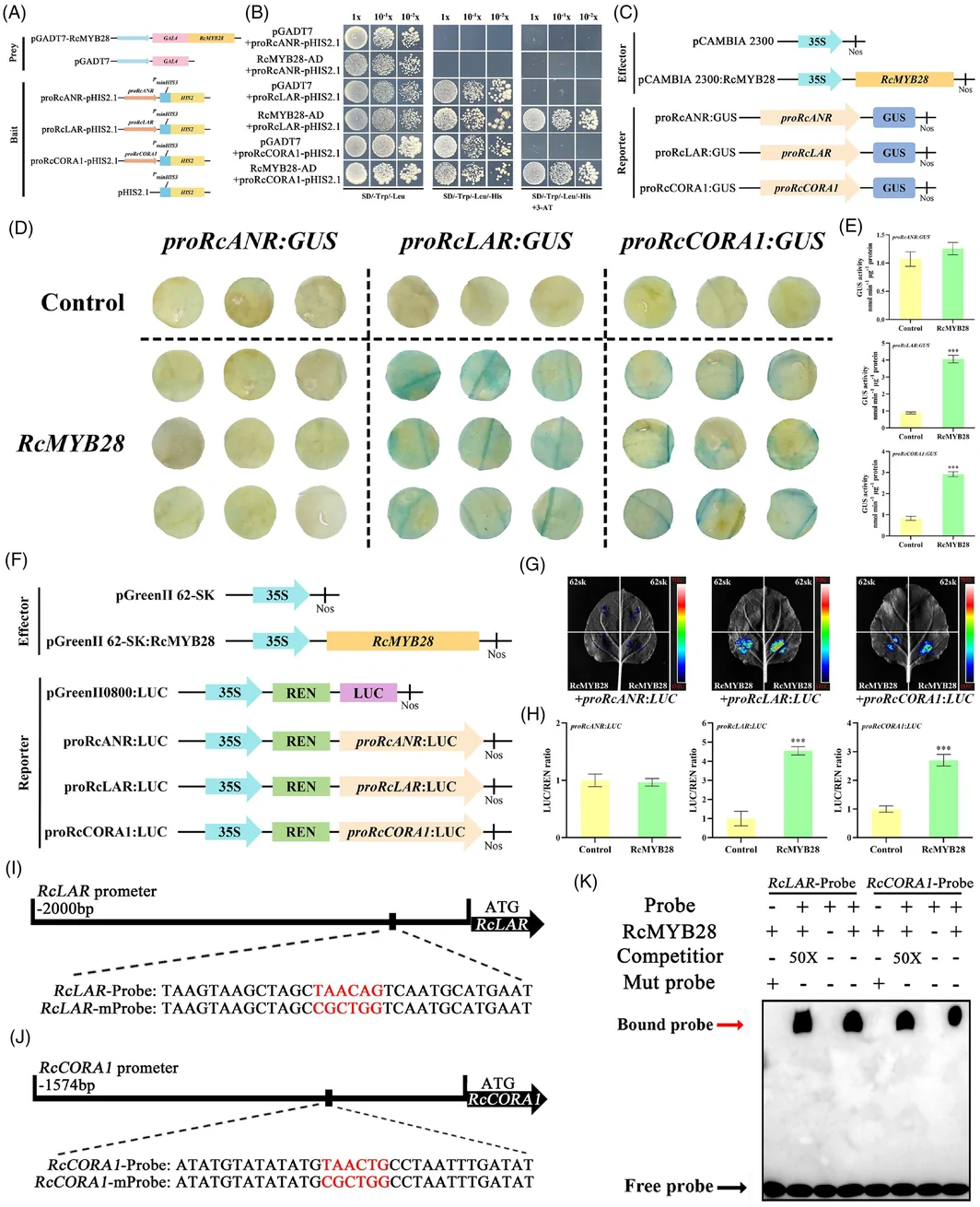
RcMYB28 binds to and activates the promoter of *RcLAR* and *RcCORA1*. (A) Schematic diagrams of constructs used for yeast one‐hybrid (Y1H) assay. (B) Growth of yeast cells co‐transformed with various bait/ prey combinations on selective media (SD/‐Trp/‐Leu, SD/‐Trp/‐Leu/‐His and SD/‐Trp/‐Leu/‐His + 3‐AT). 1×, 10^−1^× and 10^−2^× represent yeasts with original concentration (OD600 = 0.5), 10 and 100 fold dilution, respectively. (C) Schematic representation of the constructs used for GUS activity assay. The reporter construct contains the GUS driven by the promoter of *RcLAR*, *RcANR* and *RcCORA1*. The effector constructs contain *RcMYB28* driven by the CaMV 35S promoter. (D) Schematic diagram of GUS activity detected using histochemical staining in transiently expressed tobacco (*N. benthamiana*) leaves. The empty vector (pCAMBIA2300) was used as negative control. The blue color indicates activated GUS activity. (E) The GUS activity driven by *RcLAR*, *RcANR* and *RcCORA1* promoter detected by fluorometric assay and expressed as nmol 4‐methylumbelliferone μg^−1^ protein min^−1^. (F) The reporter construct contains the firefly luciferase (LUC) driven by the promoter of *RcLAR*, *RcANR* and *RcCORA1*, and the Renilla luciferase (REN) driven by the CaMV 35S promoter. The effector constructs contain *RcMYB28* driven by the CaMV 35S promoter. (G) Dual‐luciferase assay showed that *RcMYB28* promoted the expression of *RcLAR* and *RcCORA1*. (H) Transient expression assay of promoter activity, Empty effector vector (62SK) was used as the control. LUC: REN ratio of the control (tobacco leaves co‐transformed with the reporters and the empty effector vector) was taken as 1 for normalization. (I) Probes used for electrophoretic mobility shift assay. The top one is a probe that putative RcMYB28 TF‐binding element on the *RcLAR* promoter, and the bottom one has MYB cis‐element (TAACAG) mutated with CGCTGG. (J) Probes used for electrophoretic mobility shift assay. The top one is a probe that putative RcMYB28 TF‐binding element on the *RcCORA1* promoter, and the bottom one has MYB cis‐element (TAACTG) mutated with CGCTGG. (K) Electrophoretic mobility shift assay (EMSA) showing direct and specific binding of RcMYB28 to the MBS site in the promoters of *RcLAR* (left), and *RcCORA1*(right). The protein‐DNA complex and free probe are shown by arrows. + and − represent the presence or absence of the components shown on the left side. Values are mean ± SD from three independent biological replicates. Statistical significance was determined using a Student's *t*‐test (***P < 0.001).

To validate the Y1H assay results, transient expression assays were performed in tobacco leaves, and GUS activity was quantified to confirm the observed interactions (Figure 8C). Co‐infiltration of *proRcLAR*:GUS or *proRcCORA1*:GUS with 35S::*RcMYB28* in tobacco leaves resulted in deeper GUS staining, whereas leaves co‐infiltrated with 35S::*RcMYB28* and *proRcANR*:GUS showed lighter staining (Figure 8D). Moreover, the quantitative GUS activity measurements were consistent with the patterns observed in the GUS histochemical staining images (Figure 8E). To further confirm this result, luciferase reporter assays were performed in tobacco (Figure 8F). The dual‐luciferase assays revealed that RcMYB28 exhibited significant positive regulatory effects on the *RcLAR* and *RcCORA1* promoters (Figure 8G,H). However, no significant regulatory effects of RcMYB28 on the *RcANR* promoter were observed (Figure 8G,H). These results suggest that RcMYB28 acts as transcriptional activators of *RcLAR* and *RcCORA1*.

Bioinformatic analysis predicted the presence of conserved MYB cis‐acting elements within the promoter regions of *RcLAR* and *RcCORA1* (Figure 8I,J). To further validate the potential binding relationship, an electrophoretic mobility shift assay (EMSA) was performed to confirm the *in vitro* specific binding of RcMYB28 to the *RcLAR* and *RcCORA1* promoters. A strong protein‐DNA binding band was observed using the labeled oligonucleotide probe harboring TAACAG/TAACTG sequences (Figure 8K). By contrast, the formation of the complexes was completely abolished when the TAACAG/TAACTG cis‐elements were mutated to CGCTGG (Figure 8K). These competition‐binding results further verified that RcMYB28 could specifically recognize and interact with the TAACAG/TAACTG motifs located in the *RcLAR* and *RcCORA1* promoters.

### *RcCORA1* functions in drought tolerance of rose

Given that *RcCORA1* acts as a direct target of RcMYB28, we first generated transgenic rose petals overexpressing *RcCORA1* to better understand the function of *RcCORA1*. The expression level of *RcCORA1* (GenBank accession no: PZ262362) was much higher in the *RcMYB28*‐overexpression lines than in the EV lines (Figure 9A). After 24 h of dehydration stress, the petal discs from 35S:*RcCORA1* lines exhibited significantly less shrinkage compared to the EV (Figure 9B). In line with the phenotypic performance, the overexpression lines exhibited significantly lower MDA and EL levels compared with the EV control group when exposed to dehydration stress (Figure 9C,D). Meanwhile, DAB and NBT histochemical staining results showed that the petals of *RcMYB28*‐overexpressing lines had more and darker staining spots than the EV control group (Figure 9E). These data suggest that overexpression of *RcCORA1* significantly increases dehydration tolerance and facilitates ROS scavenging in plants subjected to dehydration.

**Figure 9:**
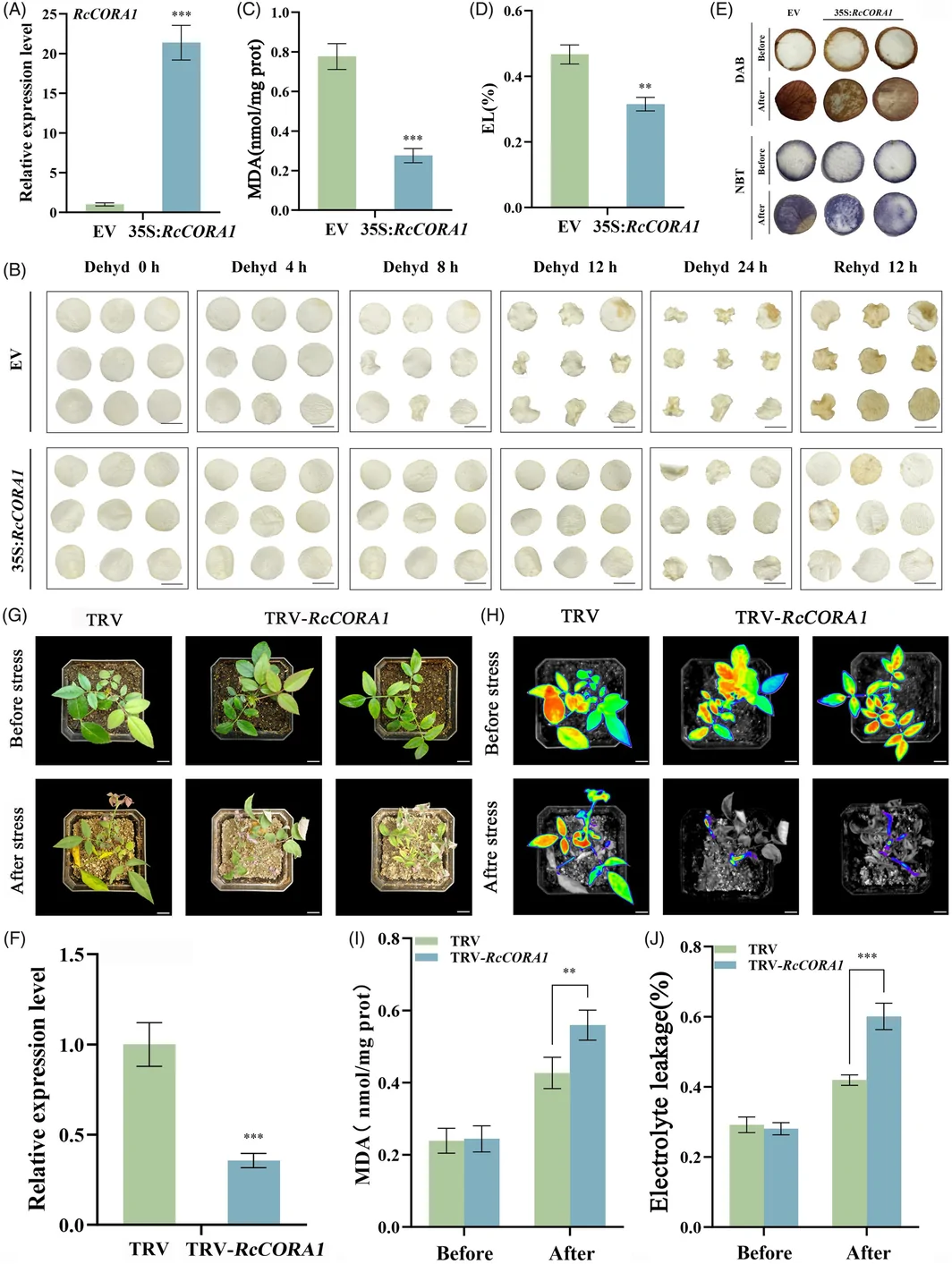
*RcCORA1* positively regulates drought tolerance. (A) Expression levels of *RcCORA1* in empty vector control (EV) and transgenic rose petals. (B) Phenotypes of *RcCORA1* transgenic rose petal discs (35S:*RcCORA1*) and empty vector control (EV) samples dehydrated for 0, 4, 8, 12, 24 h and followed by rehydration for 12 h. The rose petal discs were punched symmetrically in the middle of the petals using a 1.5‐cm hole punch. (C, D) Malondialdehyde (MDA) contents (C) and Electrolyte leakage (D) in the *RcCORA1* transgenic rose petal discs measured after dehydration. (E) *In situ* accumulation of H_2_O_2_ and O_2_^−^ of EV and *RcCORA1* transgenic rose petals, as revealed by histochemical staining with 3,3′‐diaminobenzidine (DAB) and nitrotetrazolium blue chloride (NBT), before (upper panels) and after (lower panels) dehydration. (F) qRT‐PCR analysis of *RcCORA1* in *Rosa chinensis* ‘Old blush’ silenced plants. (G) Phenotypes of virus‐induced gene silencing (VIGS) plants (TRV‐*RcCORA1*) and control plants (TRV) before and after drought. (H) Fluorescence imaging of VIGS plants (TRV‐*RcCORA1*) and control plants (TRV) before and after drought. (I, J) MDA contents (I) and Electrolyte leakage (J) in the VIGS plants (TRV‐*RcCORA1*) and control plants (TRV) before and after drought. Experiment was conducted using three biological replicates of five individual samples. Each of the samples had three technical replicates. Values are means ± SD from three independent biological replicates. Statistical significance was determined using a Student's *t*‐test (**P < 0.01, ***P < 0.001).

To further explore the role of *RcCORA1* in drought tolerance, we constructed *RcCORA1*‐knockdown rose lines via the virus‐induced gene silencing (VIGS) method. The expression level of the *RcCORA1* was significantly downregulated in the VIGS lines (designated as TRV‐*RcCORA1*) relative to the TRV control (Figure 9F). Prior to imposition of drought stress, the VIGS‐silenced plants exhibited phenotypically indistinguishable characteristics compared with the control plants (Figure 9G). However, when subjected to the drought condition, the TRV‐*RcCORA1* plants exhibited more serious wilting phenotype compared with the TRV plants (Figure 9G). Consistent with the observed phenotypic differences, the silencing of *RcCORA1* led to a significant decrease in chlorophyll fluorescence intensity, accompanied by a notable increase in both EL and MDA content in the VIGS plants relative to the TRV control group (Figure 9H–J). In conclusion, these results demonstrate that knockdown of *RcCORA1* leads to a significant impairment of drought tolerance in rose plants.

### *RcMYB28* is a downstream target of RcDREB1E

For a more comprehensive understanding of *RcMYB28* regulation, a Y1H screen was performed using the *RcMYB28* promoter as bait to identify potential TF partners from a cDNA library. Using genome annotation data from *R. chinensis* ‘Old blush’, we identified five candidate TF preys, which were subsequently screened and confirmed in this study (Table S2). Notably, transcriptome profiling of drought‐treated ‘Old Blush’ rose revealed that RcDREB1E (GenBank accession no. PZ262363) was strongly induced by drought stress (Jia et al., 2021). In line with this, *RcDREB1E* also exhibited the highest transcript abundance in rose petals subjected to dehydration treatment (Figure S9). Further sequence analysis of the RcMYB28 promoter identified two canonical DRE/CRT core motifs (site 1 and site 2), which are well‐documented cis‐acting elements specifically recognized by DREB TFs. Collectively, these findings prompted us to select RcDREB1E as the candidate prey TF for subsequent functional validation.

The interaction between RcDREB1E and the *RcMYB28* promoter was initially confirmed by a point‐to‐point Y1H assay, in which RcDREB1E was expressed as the prey and a bait construct carrying the *RcMYB28* promoter region encompassing the DRE/CRT motif was employed (Figure 10A). All of the yeast cells grew normally on SD/‐Trp/‐Leu medium. However, only the yeast cells co‐transformed with RcDREB1E prey and the bait survived on the SD/‐Trp/‐Leu/‐His and SD/‐Trp/‐Leu/‐His + 3‐AT medium (Figure 10B). The full‐length ORF of *RcDREB1E* was 729 bp, which encodes a predicted protein consisting of 242 amino acids and harbors a conserved AP2/ERF DNA‐BD (Figure S10). The phylogenetic analysis revealed that RcDREB1E shares a close evolutionary relationship with Arabidopsis DREB1A‐F, which implies that RcDREB1E belongs to the A1 subgroup (Figure S11).

**Figure 10:**
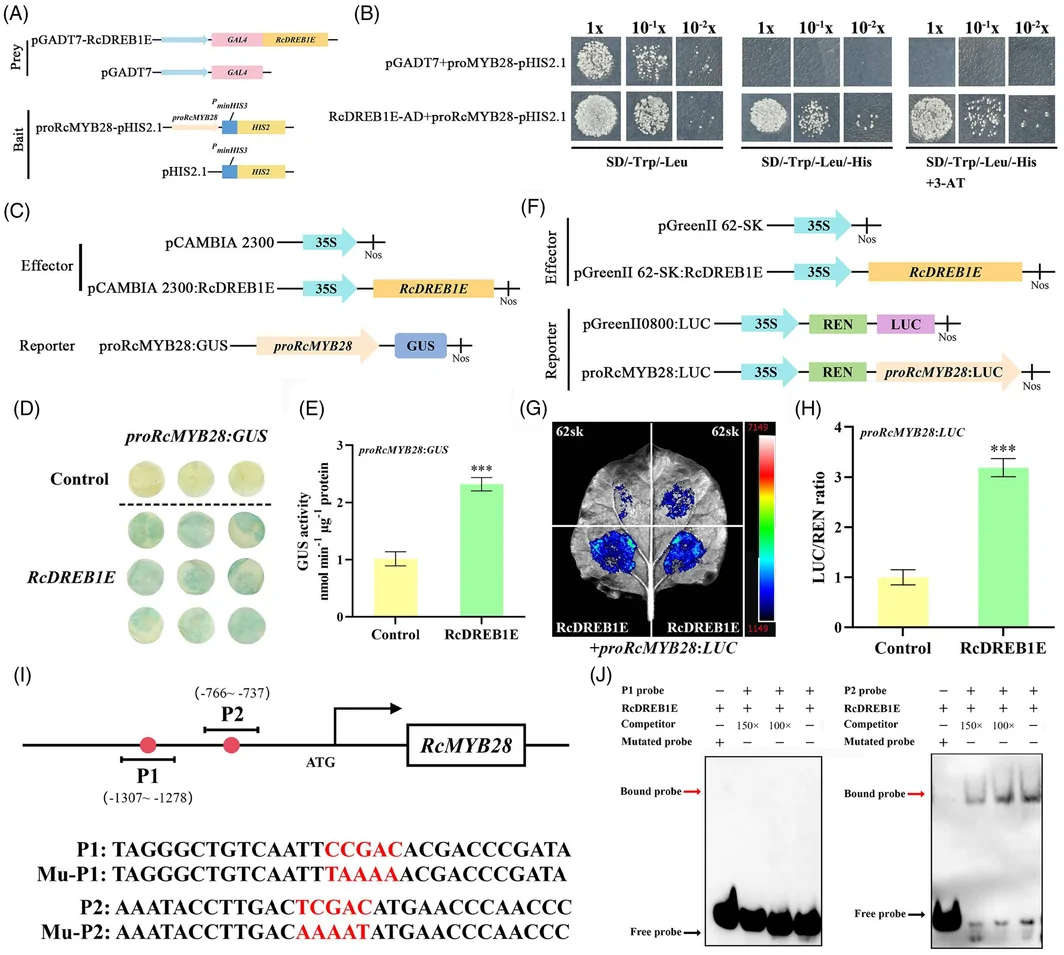
*RcMYB28* is a target gene of RcDREB1E. (A) Schematic representation of the constructs used for yeast one‐hybrid (Y1H) assay. (B) Growth of yeast cells co‐transformed with various bait/prey combinations on selective media (SD/‐Trp/‐Leu, SD/‐Trp/‐Leu/‐His and SD/‐Trp/‐Leu/‐His + 3‐AT). 1×, 10^−1^× and 10^−2^× represent yeasts with original concentration (OD_600_ = 0.5), 10 and 100 folds of dilution, respectively. (C) Schematic representation of the constructs used for GUS activity assay. The reporter construct contains the GUS driven by the promoter of *RcMYB28*. The effector constructs contain *RcDREB1E* driven by the CaMV 35S promoter. (D) Schematic diagram of GUS activity detected using histochemical staining in transiently expressed tobacco (*N. benthamiana*) leaves. The empty vector (pCAMBIA2300) was used as negative control. The blue color indicates activated GUS activity. (E) The GUS activity driven by *RcMYB28* promoter detected by fluorometric assay and expressed as nmol 4‐methylumbelliferone μg^−1^ protein min^−1^. (F) The reporter construct contains the firefly luciferase (LUC) driven by the promoter of *RcMYB28*, and the Renilla luciferase (REN) driven by the CaMV 35S promoter. The effector constructs contain *RcDREB1E* driven by the CaMV 35S promoter. (G) Dual‐luciferase assay showed that *RcDREB1E* promoted the expression of *RcMYB28*. (H) Transient expression assay of promoter activity, Empty effector vector (62SK) was used as the control. LUC: REN ratio of the control (tobacco leaves co‐transformed with the reporters and the empty effector vector) was taken as 1 for normalization. Values are means ± SD from three independent biological replicates. Statistical significance was determined using a Student's *t*‐test (***P < 0.001). (I) Schematic diagrams of the P1/P2 probe sites on the *RcMYB28* promoter. (J) EMSA assay of specific binding of RcDREB1E to the CCGAC motif sequence in *RcMYB28*. Purified His‐RcDREB1E fusion protein was incubated with a biotin‐labeled WT (Probe‐biotin) or mutated probe (mProbe‐bioton), along with or without the unlabeled competitor DNA. −, absence; +, presence.

We subsequently aimed to determine whether RcDREB1E can activate the *RcMYB28* promoter *in vivo*. To this end, we performed a GUS assay, in which RcDREB1E was used as the effector, and the reporter vector was constructed by inserting the *RcMYB28* promoter fragment containing DRE/CRT sites (Figure 10C). The GUS activity in the callus expressing *proRcMYB28*: GUS was dramatically elevated when co‐expressed with 35S:*RcDREB1E* (Figure 10D,E). Furthermore, *RcMYB28* promoter‐driven firefly luciferase (LUC) reporter constructs were developed, and a corresponding effector plasmid expressing *RcDREB1E* was generated for dual‐luciferase assays (Figure 10F). Co‐expression of the 35S:*RcDREB1E* effector and the *proRcMYB28*:LUC reporter in transient assays resulted in over a threefold increase in luciferase activity relative to the control (Figure 10G,H). Collectively, these results verified that RcDREB1E acted as a transcriptional activator of *RcMYB28*.

EMSA was employed to test the binding of RcDREB1E to the DRE/CRT cis‐acting elements *in vitro*. Incubation of the HIS‐tagged RcDREB1E protein with the labeled probes containing the P2 fragment led to the occurrence of a band shift, but not with the P1‐derived probe (Figure 10I,J). Meanwhile, when the DRE/CRT site in the P2 probes was mutated, the band shift phenomenon was also eliminated (Figure 10I,J). This confirms that RcDREB1E directly and specifically binds to the DRE/CRT site in the P2 region (Figure 10I,J). These data indicate that RcDREB1E directly binds to the *RcMYB28* promoter fragment.

### *RcDREB1E* positively modulates drought tolerance by regulating *RcMYB28* ‐mediated PAs accumulation

To investigate the functions of *RcDREB1E*, the 35S:*RcDREB1E* constructs were introduced into rose petals (Figure 11A). To visualize differences in PA accumulation, transgenic rose petals overexpressing *RcDREB1E* and EV‐transformed control petals (designated EV) were subjected to histochemical staining with the DMACA reagent. Consistent with our hypothesis, staining intensity was markedly stronger in the *RcDREB1E*‐overexpressing petals, indicating elevated PA levels relative to the EV controls (Figure 11B). Quantitative measurements showed that the levels of PAs in the *RcDREB1E* overexpression petals were significantly higher than those in the EV (Figure 11C). In addition, the *RcDREB1E* overexpression petals had significantly higher expression levels of *RhMYB28*, *RhANR*, and *RhLAR* relative to the EV (Figure 11D–F). However, RcDREB1E could not directly induce *ANR* and *LAR* expression via LUC assays (Figure S12). These data imply that *RcDREB1E* overexpression enhanced PA synthesis through directly regulating *MYB28* expression.

**Figure 11:**
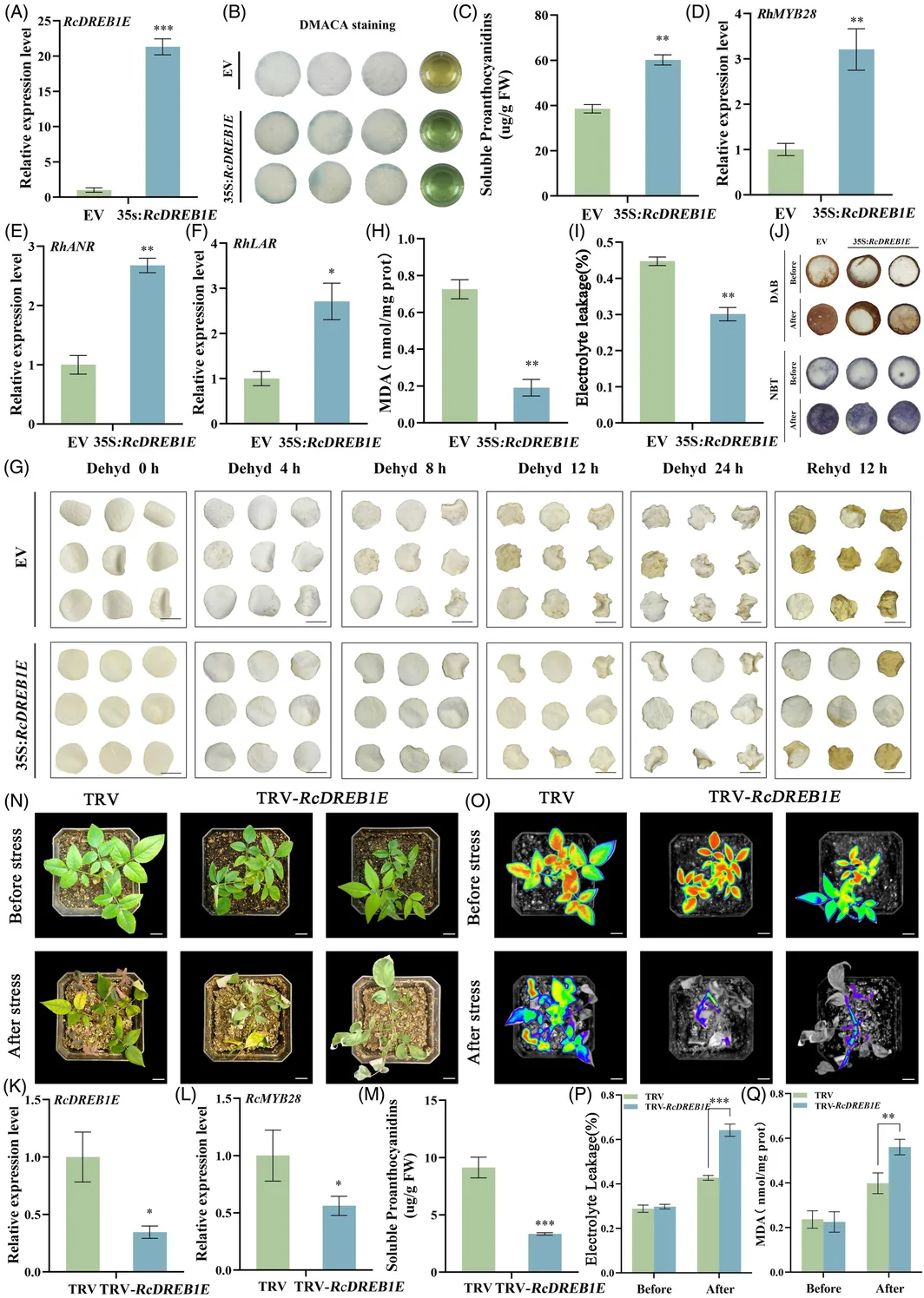
*RcDREB1E* positively regulates drought tolerance and proanthocyanidins (PAs) accumulation. (A) Expression levels of *RcDREB1E* in empty vector control (EV) and transgenic rose petals. (B) DMACA‐stained rose petals of EV and transgenic lines. (C) PA levels in the rose petals of EV and transgenic lines. (D–F) The expression levels of *RhMYB28* (D), *RhANR* (E) and *RhLAR* (F) in EV and *RcMYB28‐*overexpressing rose petals. (G) Phenotypes of *RcDREB1E* transgenic rose petal discs (35S:*RcDREB1E*) and EV control samples dehydrated for 0, 4, 8, 12, 24 h and followed by rehydration for 12 h. The rose petal discs were punched symmetrically in the middle of the petals using a 1.5‐cm hole punch. (H, I) Malondialdehyde (MDA) contents (H) and electrolyte leakage (I) in the *RcDREB1E* transgenic rose petal discs measured after dehydration. (J) *In situ* accumulation of H_2_O_2_ and O_2_^−^ of EV and *RcDREB1E* transgenic rose petals, as revealed by histochemical staining with 3,3′‐diaminobenzidine (DAB) and nitrotetrazolium blue chloride (NBT), before (upper panels) and after (lower panels) dehydration. (K, L) qRT‐PCR analysis of *RcDREB1E* (K) and *RcMYB28* (M) in *Rosa chinensis* ‘Old blush’ silenced plants. (M) PA levels in the virus‐induced gene silencing (VIGS) plants (TRV‐*RcDREB1E*) and control plants (TRV). (N) Phenotypes of VIGS plants (TRV‐*RcDREB1E*) and control plants (TRV) before and after drought. (O) Fluorescence imaging of VIGS plants (TRV‐*RcDREB1E*) and control plants (TRV) before and after drought. (P, Q) Electrolyte leakage (P) and MDA contents (Q) in the VIGS plants (TRV‐*RcDREB1E*) and control plants (TRV) before and after drought. Experiment was conducted using three biological replicates of five individual samples. Each of the samples had three technical replicates. Values are means ± SD from three independent biological replicates. Statistical significance was determined using a Student's *t*‐test (**P < 0.01, ***P < 0.001).

To further verify the role of *RcDREB1E* in dehydration tolerance, *RcDREB1E* was overexpressed in rose petals. After dehydration treatment, wilting symptoms in EV control petals were significantly more severe compared with the petals of RcMYB28 transgenic lines (Figure 11G). During dehydration treatment, EL and MDA contents were markedly reduced in the transgenic lines relative to the EV control (Figure 11H,I). Following dehydration treatment, DAB and NBT staining in the transgenic lines was substantially weaker relative to the EV control, indicating reduced ROS accumulation in these lines (Figure 11J).

A VIGS approach was employed to repress *RcDREB1E* in ‘Old blush’ to further determine the function of *RcDREB1E*. The mRNA abundance of *RcDREB1E* was significantly reduced in the silenced plants (defined as TRV‐*RcDREB1E*) relative to the TRV control (Figure 11K). Silencing of *RcDREB1E* via VIGS caused a dramatic decrease in the transcript abundance of *RcMYB28* in TRV‐*RcDREB1E* plants compared with the TRV plant (Figure 11). Consistent with this, the expression levels of two key PA biosynthetic genes, *RcANR* and *RcLAR*, were also significantly downregulated in TRV‐*RcDREB1E* lines (Figure S13). Lower PA accumulation was observed in the leaves of the TRV‐*RcDREB1E* plant than in those of the TRV (Figure 11M). Under normal growth conditions, the TRV‐*RcDREB1E* and TRV plants were morphologically similar. However, under drought stress, TRV‐*RcDREB1E* plants exhibited more severe wilting and necrosis, along with markedly higher EL and MDA levels, compared with TRV control plants (Figure 11N–Q). Together, these results indicate that *RcDREB1E* contributes to PA accumulation triggered by both short‐term dehydration shock and long‐term drought stress.

## DISCUSSION

Drought is among the most significant abiotic stresses limiting plant growth, development, geographical distribution, and crop yield (Ren et al., 2021; Yang et al., 2012). Genetic improvement aimed at developing drought‐tolerant cultivars is crucial for ensuring stable cut‐flower production and makes a substantial contribution to the sustainable development of urban gardens. To this end, a better understanding of plant responses to drought stress can provide valuable insights and genetic resources. Throughout evolution, plants have developed complex, integrated mechanisms operating at multiple biological scales to mitigate drought stress and enhance survival. Numerous studies have demonstrated that the accumulation of particular metabolites serves as a crucial adaptive mechanism, allowing plants to mitigate cellular injury caused by drought stress (Li et al., 2017, 2019). PAs, also referred to as condensed tannins, are a class of metabolites from the flavonoid biosynthetic pathway and have been demonstrated to play a direct role in protecting plant cells against abiotic stresses (Chen et al., 2025; Shen et al., 2019). However, the molecular mechanisms underlying PA accumulation in response to drought stress remain poorly understood. Therefore, elucidating the transcriptional cascade and associated regulatory modules is essential for understanding the molecular mechanisms governing PA accumulation and for developing targeted genetic strategies to enhance PA biosynthesis and improve drought tolerance.

Alongside the major enzymatic antioxidants (e.g., SOD, catalase, and glutathione peroxidase), non‐enzymatic antioxidants such as ascorbic acid and flavonoids form a core defensive system against ROS damage induced by diverse environmental stresses (Bao et al., 2025; Sun et al., 2024). Drought‐triggered accumulation of ROS in leaves has been widely reported across a range of plant species, including *Pyrus betulaefolia*, tree peony, and poplar (Niu et al., 2024; Luan et al., 2025). PAs‐mediated antioxidant defense against abiotic stress has been documented in a wide range of plant species, including *R. rugosa* under wounding and oxidative stress (Shen et al., 2019) and *Actinidia arguta* under cold stress (Sun et al., 2024). Accordingly, PAs serve as key non‐enzymatic antioxidants that scavenge excess ROS in plant tissues, effectively alleviating oxidative damage caused by adverse environmental conditions. However, whether plants actively induce PA biosynthesis in response to drought stress has not been fully characterized. Here, we demonstrate that PA accumulation in response to drought stress is a widespread phenomenon across the plant kingdom, spanning from the moss *P. acutum* to spermatophytes including ginkgo, tomato, and carnation. Although the rate and magnitude of drought‐induced PAs accumulation vary substantially across plant species, such interspecific differences are likely attributable to distinct kinetics of drought stress perception and downstream response among taxa.

TFs are known to act as key regulatory proteins that function in controlling a diversity of biological processes, including growth, development, and stress response (Dong, Chen, et al., 2024; Dong, Qi, et al., 2024; Song et al., 2024). Here, we found that the *RcMYB28* expression was upregulated under both dehydration and drought stress, suggesting that *RcMYB28* is likely related to the signal transduction triggered by these two stress conditions. In addition, *RcMYB28* is highly expressed in aerial tissues (leaves and flowers), where it positively regulates PA biosynthesis. Enhanced PA accumulation in leaves can effectively scavenge drought‐induced ROS, alleviate oxidative damage to the photosynthetic apparatus, and contribute to stomatal regulation to reduce transpirational water loss. These improvements in aerial tissue drought resistance can, in turn, reduce the physiological stress burden on roots, thereby enhancing whole‐plant drought tolerance at the systemic level. *RcMYB28*‐overexpressing tobacco and rose plants exhibited markedly enhanced dehydration tolerance, as evidenced by slower water loss rates, as well as lower MDA levels and EL, compared with WT. Drought stress is a major environmental stress well known to trigger oxidative damage in plants. Lipid peroxidation, as indicated by elevated MDA levels and increased EL, is deleterious to plant membrane integrity; accordingly, transgenic plants sustained markedly less membrane damage under drought conditions relative to the WT. Oxidative stress arising from excessive ROS accumulation constitutes the major driver of lipid peroxidation in plant cells. Drought‐mediated increase in superoxide has been observed earlier in the leaves of a number of plants (Jian et al., 2021; Peng et al., 2022). DAB and NBT staining clearly demonstrated that the transgenic lines accumulated significantly less ROS than WT under drought stress, implying that these lines experienced milder oxidative stress relative to WT plants, which was consistent with our MDA and EL measurement data. If not efficiently metabolized, ROS can inflict severe damage to plant cell membranes by oxidizing membrane‐associated biomolecules including lipids and proteins (Liu et al., 2020; Zhu et al., 2018). Given that ROS accumulation under abiotic stress is primarily governed by the balance between ROS production and scavenging, the reduced ROS levels observed in the transgenic lines strongly suggest that their antioxidant scavenging systems function with higher efficiency relative to WT controls. In addition to the well‐characterized major enzymatic antioxidants (including SOD, catalase, and glutathione peroxidase), non‐enzymatic metabolites including PAs, ascorbic acid, and polyamines serve as core non‐enzymatic antioxidants that mitigate ROS‐mediated oxidative damage triggered by diverse environmental stresses (Li et al., 2017; Liu et al., 2023; Shen et al., 2019). *RcANR* and *RcLAR*, two key enzymes of PAs biosynthesis, the expression of those genes of in *RcMYB28* overexpression plants were higher than that of the WT. However, it has to be mentioned that RcMYB28 directly activates on *RcLAR* promoter, not on *RcANR* promoter, and it different from MsMYB12 in *Malus sieversii*, as MsMYB12 could activates both on the promoters of *MsANR* and *MsLAR* (Wang, Ran, et al., 2017; Wang, Xu, et al., 2017).

To adapt to adverse environmental stresses, plants employ dynamic regulation of a broad repertoire of stress‐responsive genes, which constitutes a core molecular mechanism underlying plant stress response and acclimation (Liu et al., 2025; Zhao et al., 2025). To further dissect the regulatory role of *RcMYB28* and clarify the molecular mechanism underlying its mediated enhancement of drought tolerance, we quantified the transcript abundance of a panel of stress‐responsive genes under the drought treatment. These selected genes are implicated in ascorbic acid biosynthesis (*DHAR*, *MDHAR*), cellular water retention (*P5CS*, *LEA5*), and osmotic homeostasis adjustment (*CORA1*, *PIP2;1*), and these genes or their homologs in other plant species have been widely reported to function in abiotic stress response. qRT‐PCR analysis demonstrated that the transcript levels of these genes were markedly upregulated in the transgenic lines relative to WT controls under drought stress. This finding aligns with previous reports that overexpression of a TF can activate a cohort of target genes, which act synergistically to mitigate the detrimental effects of abiotic stresses (Zhang et al., 2022). While the expression of all assayed genes were upregulated in response to drought stress, they remained markedly higher in the transgenic lines relative to WT controls, demonstrating that these genes were more potently induced by drought in the *RcMYB28*‐overexpressing plants. Of note, the transcript abundance of *CORA1* was significantly higher than that of all other assayed stress‐responsive genes, making it the most strongly induced candidate among the tested genes. We hypothesized that *RcMYB28* might directly induce the *RcCORA1* expression. Y1H, GUS assay and LUC reporter system were further verified that *RcMYB28* could activate the expression of *RcCORA1*. Notably, no previous studies have reported the direct transcriptional regulation of *CORA* genes by MYB family TFs. Thus, our characterization of *RcCORA1* as a direct downstream target of RcMYB28 provides critical new insights into the MYB‐mediated regulatory network underlying plant abiotic stress responses. Overexpression of *RcCORA1* in rose petals led to enhanced dehydration tolerance, whereas knockdown of *RcCORA1* significantly compromised drought tolerance capacity, demonstrating that *RcCORA1* plays a critical role in regulating drought tolerance in rose. Therefore, we propose that *RcMYB28* functions in orchestrating the drought tolerance response in rose, at least in part, by promoting ROS scavenging via regulating *RcCORA1* expression and PA accumulation.

Accumulating evidence has demonstrated that DREB family TFs, which are phylogenetically divided into the A‐1 to A‐6 subgroups, play critical regulatory roles in plant responses to diverse abiotic stresses (Geng et al., 2023) DREBs function in abiotic stresses response by binding to the DRE/CRT elements in their target gene promoters (Chen et al., 2024). Although the promoter of *RcMYB28* contains two DRE/CRT elements, the DRE/CRT element in the P2 region was recognized by RcDREB1E via Y1H and EMSA assays. It implies that *RcMYB28* may serve as a target gene of RcDREB1E. Additionally, GUS and LUC assays indicated that RcDREB1E could directly activate the promoter of *RcMYB28* by interacting with the CCGAC element. Relative to EV controls, *RcDREB1E* overexpression significantly increased *RcMYB28* and PA levels in rose petals and enhanced dehydration tolerance, whereas silencing of *RcDREB1E* had the opposite effect. Therefore, our findings demonstrate that RcDREB1E functions as a critical positive regulator of drought stress tolerance in rose, which exerts its regulatory function by directly binding to the DRE/CRT cis‐element in the *RcMYB28* promoter to transcriptionally activate its expression. Collectively, these results demonstrate that DREB family TFs function in a hierarchical drought stress regulatory network: they not only directly control the expression of functional genes that mitigate drought‐induced damage to plant cells, but also regulate downstream stress‐responsive TFs. In *Lilium lancifolium*, LlR3MYB‐mediated flavonoid biosynthesis confers cold stress tolerance through the LlDREB‐LlCHS2 regulatory cascade (Yong et al., 2026). In contrast, we demonstrate that the RcDREB1E‐RcMYB28 module in rose specifically targets the late PA biosynthetic gene (*RhLAR*) under drought stress, rather than early flavonoid genes or other flavonoid sub‐branches. This target specificity reflects a functional specialization of the module for PA‐dependent drought tolerance in rose. This supports the conclusion that the RcDREB1E‐RcMYB28 regulatory module acts as a key signaling node to relay drought stress signals to downstream targets, and thus orchestrates the global drought stress response in rose.

Here, we present a working model depicting the regulatory mechanism of *RcMYB28* in mediating drought stress responses in rose (Figure 12). Drought stress triggers the *RcDREB1E* activation. RcDREB1E, upon activation, operates upstream of *RcMYB28*: it binds to DRE/CRT cis‐elements in the *RcMYB28* promoter to initiate its expression, which is then directly upregulated by *RcMYB28* for *RcLAR* and *RcCORA1*. Upregulation of the RcMYB28‐RcLAR/RcCORA1 module promotes stress responses and PAs synthesis to alleviate drought‐associated damage by maintaining ROS homeostasis and osmotic adjustment. In summary, these results not only provide novel insights into the regulatory roles and physiological functions of MYB family TFs in drought stress tolerance, but also lay a theoretical foundation for the genetic improvement of drought resistance in rose and other ornamental plants.

**Figure 12:**
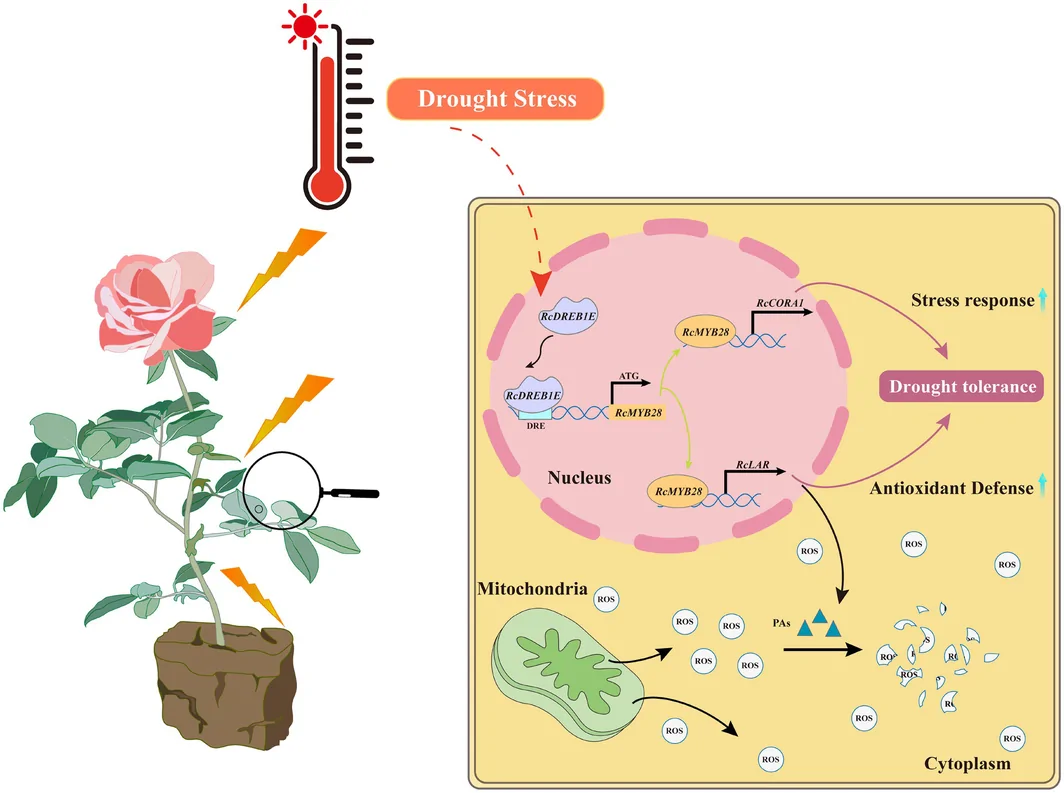
A proposed model for the regulatory function of RcDREB1E‐RcMYB28 in response to drought stress. Drought stress activates the expression of *RcDREB1E*. RcDREB1E then binds to the DRE/CRT element in the promoter of *RcMYB28*, which, in turn, regulates *RcLAR* and *RcCORA1* genes, leading to enhanced drought tolerance in modulation of proanthocyanidins (PAs) accumulation.

## MATERIALS AND METHODS

### Plant material and stress treatments

Eighteen plant species spanning bryophytes, ferns, gymnosperms, and angiosperms, namely *P. acutum*, *H. plumaeforme*, *W. japonica*, *D. erythrosora*, *G. biloba*, *T. baccata*, *M. denudata*, *M. sylvestris*, *O. speciosa*, *H. macrophylla*, *O. articulata*, *P. fruticosa*, *A. frutescens*, *R. simii*, *H. annuus*, *I. hawkeri*, *P. atkinsiana*, and *G. tenera*, were cultivated under natural conditions in the Botanical Garden of Donghu Campus, Zhejiang A&F University. *Solanum lycopersicum*, *D. caryophyllus*, *P. amabilis* and *H.vittatum* were maintained in the greenhouse of Zhejiang A&F University. For the dehydration treatment, fully opened petals were used as experimental material for all species, with the exception of *P. acutum*, *H. plumaeforme*, *W. japonica*, *D. erythrosora*, *G. biloba*, *T. baccata*, and *M. denudata*, for which mature leaves were collected instead. Specifically, samples were placed on filter paper and subjected to dehydration at room temperature. Samples were harvested at 0, 30, 60, and 90 min after the onset of dehydration, respectively, and the PAs content was subsequently quantified.

Mature seeds of *R. chinensis* ‘Old Blush’ (self‐pollinated progeny sourced from the Germplasm Resource Garden, Zhejiang Agriculture and Forestry University) were subjected to a 60‐day stratification treatment in wet sand at 4°C. Following germination, seedlings were transferred to soil pots and maintained in an artificial climate chamber set to a 16 h light/8 h dark cycle, with a day/night temperature regime of 22°C/20°C. Three‐month‐old seedlings were utilized to investigate *RcMYB28* expression profiles in response to dehydration and salt stress. Seedlings were subjected to dehydration by placing them on dry filter paper at 22°C for 0, 0.5, 1, or 1.5 h to assess time‐dependent responses. For salt treatment, seedlings were treated with 150 mM NaCl by immersing them in conical flasks for 0, 1, 5, 12, 24, or 48 h. The leaf samples were collected at the designated time points after treatment. Roots, stems, leaves, flowers, and fruits were harvested from 2‐year‐old ‘Old Blush’ for tissue‐specific expression analysis.

### RNA extraction and qRT‐PCR

Total RNA was isolated using a modified CTAB method (Li et al., 2008). qRT‐PCR was conducted on the ABI 7500 Real‐Time System (Applied Biosystems, Foster City, CA, US) using the SYBR Premix Ex Taq (Takara, Dalian, China) according to the manufacturer's instructions. *EF1α* and *GAPDH* were used as internal reference genes for rose and tobacco, respectively (Chen et al., 2025). The 2^−ΔΔCt^ method was conducted to calculate relative expression levels (Livak & Schmittgen, 2001), and each data set was based on three independent biological replicates. Details of the primers employed are provided in Table S1.

### Cloning and sequence analysis

The full‐length CDS of *RcMYB28* (LOC112171864) was isolated from *R. chinensis* leaf cDNA according to the previously reported transcriptome data of drought‐treated rose seedlings (Jia et al., 2021). Phylogenetic relationships were inferred by the neighbor‐joining (NJ) method with 1000 bootstrap replicates in MEGA X, and multiple sequence alignments were constructed using Clustal X (version 1.81).

### Subcellular localization analysis

The coding sequence of *RcMYB28* was amplified and directionally cloned into the pLGFP1301 vector in fusion with the green fluorescent protein (GFP) reporter, yielding the 35S:*RcMYB28*‐GFP construct. The recombinant plasmid or the EV (35S:GFP) mixed with VirD2NLS‐mCherry was co‐transformed into tobacco (*Nicotiana benthamiana*) leaves as described previously (Gong et al., 2015). Fluorescence signals were visualized and imaged with a confocal laser scanning microscope (Zeiss LSM 510 Meta, Oberkochen, Germany).

### Transcriptional activation assays

The complete *RcMYB28* coding sequence (dFL) and two deletion derivatives (dΔN and dΔC) were inserted into the pGBKT7 bait vector (Clontech, Mountain View, CA, USA), which were transformed into yeast strain AH109. Yeast transformants were incubated on SD/‐Trp plates or on SD/‐Trp/‐His/‐Ade plates, supplemented with or without X‐α‐gal, at 30°C for 3 days.

### Vector construction and plant transformation

The full‐length CDS of *RcMYB28* was ligated into pCAMBIA 2300 (Cambia, Canberra, Australia), yielding the 35S:*RcMYB28* construct. The resulting vector was transformed into *Agrobacterium tumefaciens* strain EHA105 and then carried out for transformation of tobacco (*N. tabacum*) and rose somatic embryos (*R. rugosa* ‘Bao white’) according to the previous reports (Ning et al., 2012; Xing et al., 2014). Somatic embryos were successfully initiated from young leaves of *R. rugosa* ‘Bao white’ and could be maintained through continuous proliferation, enabling long‐term preservation (Xing et al., 2014). The abundance of *RcMYB28* transcripts in the transgenic lines was measured by qRT‐PCR. The two transgenic tobacco lines at T2 generation and two transgenic rose lines with high expression of *RcMYB28* were used for the subsequent experiments.

### Transient overexpression in rose petals

Transient expression in rose petal discs was achieved by resuspending transformed *Agrobacterium* cultures in infiltration buffer to an OD_600_ of 1.0 (He et al., 2023). The petal discs were immersed in the suspension and infiltrated under a vacuum at 0.07–0.08 MPa for 5 min, repeated twice (Luo et al., 2024). Three experimental replicates were set up for each treatment, and approximately 200 petal discs were sampled from six flowers.

### Virus‐induced gene silencing

Partial cDNA fragments of *RcCORA1* and *RcDREB1E* were isolated and ligated into the pTRV2 vector, respectively. The pTRV1, pTRV2 (control), and fusion constructs (TRV‐*RcCORA1* and TRV‐*RcDREB1E*) were individually introduced into *A. tumefaciens* strain GV3101. For VIGS assay, transfection of 30 days rose cutting was conducted by incubating in the bacterial suspension of pTRV1, pTRV2 or fusion constructs (v:v, 1:1) according to the previous reports (Cao et al., 2019; Tian et al., 2014). Cuttings were infiltrated under vacuum and then transplanted into soil pots, where they were kept in a controlled‐environment growth chamber set to a 16 h light/8 h dark cycle and a day/night temperature regime of 22°C/20°C. Four weeks later, leaf tissue was collected from the resulting seedlings and subjected to qRT‐PCR analysis to confirm silencing efficiency for further study.

### Drought tolerance assays

For dehydration analysis, 20‐day‐old *in vitro*‐grown tobacco seedlings of overexpression lines were placed on filter paper and subjected to dehydration for a maximum of 70 min. FW was recorded at 10‐min intervals to determine the relative water loss rate based on the initial value. For the drought tolerance assay, 30‐day‐old transgenic tobacco plants grown in soil were subjected to water deprivation for 15 days, followed by recovery under regular irrigation for 3 days. For rose petal discs with transient overexpression, the discs were subjected to dehydration for 24 h and subsequently rehydrated in deionized water for 12 h. After 24 h of dehydration, petal disc samples were harvested to determine the overexpression efficiency by qRT‐PCR. As for the VIGS rose plants, 40‐day‐old rose cutting seedlings were exposed to drought treatment for 14 days. In another experiment, 30‐day‐old *R. rugosa* overexpression lines (OE‐2 and OE‐4) and WT plants were exposed to withholding water for 15 days and then re‐watered for 3 days.

### Physiological measurements and histochemical staining

The contents of MDA, hydrogen peroxide (H_2_O_2_), superoxide anion (O_2_^−^), and the activities of antioxidant enzymes (POD and SOD) were determined with specific detection kits, following the manufacturer's protocols (Jiancheng, Nanjing, China). The level of EL was measured following previously reported protocols (Feng et al., 2021). DAB and NBT histochemical staining were used to determine the accumulation of H_2_O_2_ and O_2_^−^, respectively (Bai et al., 2023).

### Staining and determination of PAs

The rose somatic embryos were stained with 0.2% dimethylaminocinnamaldehyde (DMACA) solution (methanol:6 m HCl = 1:1, v/v) for 10 min, followed by three washes with 70% ethanol to visualize PA accumulation. PAs determination was performed according to the previously reported method (Luo et al., 2016). In brief, frozen tissues were pulverized in liquid nitrogen and subsequently extracted in 3 ml of a solvent mixture containing 70% acetone, 29.5% distilled water, and 0.5% acetic acid (v/v) for 24 h at 4°C under dark conditions. Following centrifugation at 12 879 *g* and 4°C for 10 min, the supernatant was collected and reacted with 2.5 ml of DMACA reagent (0.1% DMACA, 90% ethanol, 10% HCl) for 30 min at room temperature. The absorbance readings at 640 nm were quantified as PAs concentrations by reference to a standard curve generated with (+)‐catechin (Sigma‐Aldrich, St. Louis, MO, USA https://www.sigmaaldrich.com/).

### Library screening and Y1H assay

The promoter fragment of *RcMYB28* was amplified from ‘Old blush’ and cloned into the pHIS2.1 vector. Following integration of the pHIS2.1‐*proRcMYB28* construct into yeast strain YM4271 (Clontech, CA, USA), positive colonies were selected on SD/‐His/‐Ura plates at 30°C. This bait strain was then employed to screen a *R. chinensis* ‘Old blush’ cDNA library (Clontech, CA, USA). Individual colonies were picked, PCR‐amplified, and sequenced to identify TFs involved in the interaction.

The ORFs of *RcMYB28* and *RcDREB1E* were amplified and then ligated into the pGADT7 vector, creating the prey constructs. Promoter regions of *RcANR*, *RcLAR*, and *RcCORA1* were individually cloned upstream of the minimal *HIS3* promoter in the pHIS2.1 vector to generate corresponding bait constructs. Plasmid pairs were co‐transformed into yeast Y187, and interaction screening was performed on SD/‐Trp/‐Leu/‐His plates containing the optimal concentration of 3‐amino‐1,2,4‐triazole (3‐AT) determined for each bait–prey pair.

### Dual‐luciferase assay

The promoters of *RcANR*, *RcLAR*, *RcCORA1*, and *RcMYB28* were inserted upstream of the luciferase reporter cassette in the pGreenII 0800‐LUC vector, generating reporter constructs. Correspondingly, the full‐length CDSs of *RcMYB28* and *RcDREB1E* were cloned into the pGreenII 62‐SK vector to create effector constructs for transient expression assays. Subsequently, these were transformed into the *A. tumefaciens* strain GV3101 (pSoup). The reporter and effector vectors (mixed in a 1:5 ratio) were infiltrated into the leaves of the 20 days old tobacco (*N. benthamiana*) as described by Yang et al. (2025). Firefly (LUC) and *Renilla* (REN) luciferase activities were quantified using the Dual‐Luciferase Reporter Assay System (Promega, Madison, WI, USA) for each sample. The transactivation was calculated as the ratio of LUC/REN activity.

### GUS reporter assay

The promoter sequences of *RcANR*, *RcLAR*, *RcCORA1*, and *RcMYB28* were inserted into a pCAMBIA1305.1 vector to generate report constructs, whereas the CDSs of *RcDREB1E* and *RcMYB28* were ligated into a pCAMBIA 2300 vector to obtain effector constructs. The recombinant vectors described above were separately introduced into *A. tumefaciens* strain GV3101 and subsequently co‐infiltrated into *N. benthamiana* leaves following established protocols (Luo et al., 2024). The infiltrated leaf discs were immersed in GUS staining solution (2 mM X‐Gluc, 100 mM sodium phosphate buffer) for 24 h at 37°C and then washed with 75% ethanol. The GUS activity was determined using specific kits (Cat. SL7161; Coolaber, Beijing, China).

### Electrophoretic mobility shift assay

The full‐length CDSs of *RcDREB1E* and *RcMYB28* were separately inserted into the pET‐28A vector to generate a recombinant vector with a His‐tag fusion, followed by transformation into *Escherichia coli* strain BL21 (DE3) (Dong, Chen, et al., 2024; Dong, Qi, et al., 2024). Proteins were expressed by induction with 1 mM IPTG (isopropyl β‐d‐1‐thiogalactopyranoside) at 16°C for 24 h, after which the target proteins were affinity‐purified on Ni‐NTA agarose (Qiagen, Hilden, Germany) following the supplier's instructions. Biotin‐labeled DNA probes, including those containing the original sequence and the mutated sequence, were synthesized by Aiki Baker Biological Company (Wuhan, China). The LightShift Chemiluminescent EMSA Kit (Thermo Fisher Scientific, Waltham, MA, USA) was employed for EMSA analyses, following the supplier's instructions. Reactions containing probes and fusion proteins (20 μl total volume) were incubated with or without competitor oligonucleotides. Following electrophoresis on 6% polyacrylamide gels, protein‐DNA complexes were transferred to nylon membranes, cross‐linked by UV irradiation, and visualized using chemiluminescent detection as previously described (Wang et al., 2021).

### Statistical analysis

Each treatment and genotype was represented by three independent biological replicates. Data were subjected to one‐way anova, and means were compared using Fisher's LSD test. Significance levels are denoted by asterisks: *P < 0.05, **P < 0.01, and ***P < 0.001.

## AUTHOR CONTRIBUTIONS

PL and YS conceived and designed this research. YZ, HZ, and LC performed most of the experiments. YZ and HZ performed the *Agrobacterium*‐mediated transformation of tobacco and rose. LC conducted the Y2H and BiFC assays. YC performed Y1H and LUC assays. JX, WC, and YC conducted English editing. YZ, HZ, and LC analyzed the data and wrote the manuscript. All authors read and approved the final manuscript.

## CONFLICT OF INTEREST

The authors declare no conflicts of interest.

## Supporting information


**Figure S1.** qRT‐PCR analysis of *RhANR* and *RhLAR* in the rose (*Rosa hybrida*) petals and leaves during the dehydration treatment.
**Figure S2.** Heatmap analysis of differentially expressed MYB transcription factors under drought stress at 0, 30, and 60 days.
**Figure S3.** Phylogenetic tree constructed based on the differentially expressed MYB transcription factors of ‘Old blush’ under drought stress and MYB proteins from *Arabidopsis thaliana*.
**Figure S4.** Multiple alignments of RcMYB28 and MYBs from other plants.
**Figure S5.** Phenotypic observation of *RcMYB28*‐overexpressing tobacco leaves.
**Figure S6.** qRT‐PCR analysis of *NtANS* and *NtUFGT* in the *RcMYB28*‐overexpressing tobacco lines.
**Figure S7.** Multiple sequence alignment of RcMYB28 from *Rosa chinensis* and RrMYB28 from *Rosa rugosa*.
**Figure S8.** qRT‐PCR analysis of genes implicated in stress response in the *RcMYB28‐*overexpressing rose lines (*R. rugosa*) after drought.
**Figure S9.** Expression patterns of *RcDREB1E* under dehydration treatment.
**Figure S10.** Multiple alignments of RcDREB1E and DREBs from other plants.
**Figure S11.** Phylogenetic tree constructed based on amino acids of RcDREB1E (marked with pentagram) and DREBs of other plants.
**Figure S12.** Effects of RcDREB1E on the promoter activity of *RcLAR* and *RcANR* via Dual‐LUC assays.
**Figure S13.** qRT‐PCR analysis of *RcANR* and *RcLAR* in the TRV*‐RcDREB1E* seedlings.
**Table S1.** Primer sequences used in this study.
**Table S2.** Information about the RcMYB28 promoter‐interacting proteins.

## Data Availability

Data that support the findings of this study are available within the article and its Supporting Information files.

## References

[tpj71169-bib-0001] An, J.P. , Li, R. , Qu, F.J. , You, C.X. , Wang, X.F. & Hao, Y.J. (2018) R2R3‐MYB transcription factor *MdMYB23* is involved in the cold tolerance and proanthocyanidin accumulation in apple. The Plant Journal, 96, 562–577. Available from: 10.1111/tpj.14050 30054966

[tpj71169-bib-0002] Bai, D. , Gu, S. , Qi, X. , Sun, L. , Lin, M. , Wang, R. et al. (2023) The ERF‐VII transcription factor *AvERF75* positively regulates the waterlogging tolerance by interacting with *AvLOB41* in kiwifruit (*Actinidia valvata*). Environmental and Experimental Botany, 212, 105401. Available from: 10.1016/J.ENVEXPBOT.2023.105401

[tpj71169-bib-0003] Bao, H.H. , Yuan, L. , Luo, Y.C. , Zhang, J.X. , Liu, X. , Wu, Q.J. et al. (2025) The transcription factor WRKY41‐FLAVONOID 3'‐HYDROXYLASE module ffne‐tunes ffavonoid metabolism and cold tolerance in potato. Plant Physiology, 197, kiaf070. Available from: 10.1093/plphys/kiaf070 PMC11879589 39977116

[tpj71169-bib-0004] Cao, X. , Yan, H. , Liu, X. , Li, D. , Sui, M. , Wu, J. et al. (2019) A detached petal disc assay and virus‐induced gene silencing facilitate the study of *Botrytis cinerea* resistance in rose flowers. Horticulture Research, 6, 136. Available from: 10.1038/s41438-019-0219-2 PMC6885046 31814989

[tpj71169-bib-0005] Chen, C. , Zhou, X. , Cao, B. , Feng, S. , Bin, T. , Zhang, Y. et al. (2025) The transcription factor *MYB8* positively regulates flavonoid biosynthesis of *Scutellaria baicalensis* in response to drought stress. Plant, Cell & Environment, 48, 8898–8914. Available from: 10.1111/pce.70178 40931714

[tpj71169-bib-0006] Chen, L.N. , Chen, Y.M. , Zhang, H.Y. , Shen, Y. , Cui, Y.Y. & Luo, P. (2024) *ERF54* regulates cold tolerance in *Rosa multiflora* through DREB/COR signalling pathways. Plant, Cell and Environment, 47, 1185–1206. Available from: 10.1111/pce.14796 38164066

[tpj71169-bib-0007] Dai, W. , Wang, M. , Gong, X. & Liu, J.H. (2018) The transcription factor *fc WRKY40* of *Fortunella crassifolia* functions positively in salt tolerance through modulation of ion homeostasis and proline biosynthesis by directly regulating *SOS2* and *P5CS1* homologs. New Phytologist, 219, 972–989. Available from: 10.1111/nph.15240 29851105

[tpj71169-bib-0008] Dong, D. , Qi, C. , Zhang, J. , Deng, Q. , Xia, P. , Li, P. et al. (2024) *CsHSFA1d* promotes drought stress tolerance by increasing the content of raffinose family oligosaccharides and scavenging accumulated reactive oxygen species in cucumber. Plant and Cell Physiology, 65, 809–822. Available from: 10.1093/pcp/pcae023 38564325

[tpj71169-bib-0009] Dong, H. , Chen, Q. , Fu, Y. , Xie, H. , Li, T. , Li, D. et al. (2024) *PbGBF3* enhances salt response in pear by upregulating *PbAPL2* and *PbSDH1* and reducing ABA‐mediated salt sensitivity. The Plant Journal, 119, 2837–2853. Available from: 10.1111/tpj.16953 39073914

[tpj71169-bib-0010] Dubos, C. , Stracke, R. , Grotewold, E. , Weisshaar, B. , Martin, C. & Lepiniec, L. (2010) MYB transcription factors in *Arabidopsis* . Trends in Plant Science, 15, 573–581. Available from: 10.1016/j.tplants.2010.06.005 20674465

[tpj71169-bib-0012] Feng, Y. , Wang, Y. , Zhang, G. , Gan, Z. , Gao, M. , Lv, J. et al. (2021) Group‐C/S1 bZIP heterodimers regulate *MdIPT5b* to negatively modulate drought tolerance in apple species. The Plant Journal, 107, 399–417. Available from: 10.1111/tpj.15296 33905154

[tpj71169-bib-0013] Geng, L. , Ren, J. , Ji, X. , Yan, S. & Song, X.S. (2023) Over‐expression of *DREB46* enhances drought tolerance in *Populus trichocarpa* . Journal of Plant Physiology, 281, 153923. Available from: 10.1016/j.jplph.2023.153923 36657232

[tpj71169-bib-0014] Gong, X. , Zhang, J. , Hu, J. , Wang, W. , Wu, H. , Zhang, Q. et al. (2015) FcWRKY70, a WRKY protein of *Fortunella crassifolia*, functions in drought tolerance and modulates putrescine synthesis by regulating arginine decarboxylase gene. Plant, Cell and Environment, 38, 2248–2262. Available from: 10.1111/pce.12539 25808564

[tpj71169-bib-0015] He, G. , Zhang, R. , Jiang, S. , Wang, H. & Ming, F. (2023) The MYB transcription factor *RcMYB1* plays a central role in rose anthocyanin biosynthesis. Horticulture Research, 10, uhad080. Available from: 10.1093/hr/uhad080 PMC10261888 37323234

[tpj71169-bib-0016] He, Y. , Li, W. , Lv, J. , Jia, Y. , Wang, M. & Xia, G. (2012) Ectopic expression of a wheat MYB transcription factor gene, *TaMYB73*, improves salinity stress tolerance in *Arabidopsis thaliana* . Journal of Experimental Botany, 63, 1511–1522. Available from: 10.1093/jxb/err389 22140235

[tpj71169-bib-0017] Jia, X. , Feng, H. , Bu, Y. , Ji, N. , Lyu, Y. & Zhao, S. (2021) Comparative transcriptome and weighted gene co‐expression network analysis identify key transcription factors of *Rosa chinensis* ‘old blush’ after exposure to a gradual drought stress followed by recovery. Frontiers in Genetics, 12, 690264. Available from: 10.3389/fgene.2021.690264 PMC8320538 34335694

[tpj71169-bib-0018] Jian, W. , Zheng, Y. , Yu, T. , Cao, H. , Chen, Y. , Cui, Q. et al. (2021) *SlNAC6*, a NAC transcription factor, is involved in drought stress response and reproductive process in tomato. Journal of Plant Physiology, 264, 153483. Available from: 10.1016/j.jplph.2021.153483 34371311

[tpj71169-bib-0019] Khan, M. , Hu, J. , Dahro, B. , Ming, R. , Zhang, Y. , Wang, Y. et al. (2021) *ERF108* from *Poncirus trifoliata* (L.) Raf. functions in cold tolerance by modulating raffinose synthesis through transcriptional regulation of *PtrRafS* . The Plant Journal, 108, 705–724. Available from: 10.1111/tpj.15465 34398993

[tpj71169-bib-0020] Li, K. , Xing, C. , Yao, Z. & Huang, X. (2017) PbrMYB21, a novel MYB protein of *Pyrus betulaefolia*, functions in drought tolerance and modulates polyamine levels by regulating arginine decarboxylase gene. Plant Biotechnology Journal, 15, 1186–1203. Available from: 10.1111/pbi.12708 PMC5552480 28190292

[tpj71169-bib-0021] Li, W. , Fu, L. , Geng, Z. , Zhao, X. , Liu, Q. & Jiang, X. (2021) Physiological characteristic changes and full‐length transcriptome of rose (*Rosa chinensis*) roots and leaves in response to drought stress. Plant & Cell Physiology, 61, 2153–2166. Available from: 10.1093/pcp/pcaa137 33165546

[tpj71169-bib-0022] Li, W. , Geng, Z. , Zhang, C. , Wang, K. & Jiang, X. (2021) Whole‐genome characterization of *Rosa chinensis* AP2/ERF transcription factors and analysis of negative regulator *RcDREB2B* in *Arabidopsis* . BMC Genomics, 22, 90. Available from: 10.1186/s12864-021-07396-6 PMC7844920 33509074

[tpj71169-bib-0023] Li, Z. , Chen, W. , Zhang, C. , Du, C. , Shao, G. , Cui, Y.Y. et al. (2019) *RcMYBPA2* of *Rosa chinensis* functions in proanthocyanidin biosynthesis and enhances abiotic stress tolerance in transgenic tobacco. Plant Cell, Tissue and Organ Culture, 137, 441–454. Available from: 10.1007/s11240-019-01580-z

[tpj71169-bib-0024] Li, Z. , Liu, G. , Zhang, J. , Zhang, J. & Bao, M. (2008) Extraction of high‐quality tissue‐specific RNA from London plane trees (*Platanus acerifolia*), permitting the construction of a female inflorescence cDNA library. Functional Plant Biology, 35, 159–165. Available from: 10.1071/FP07212 32688767

[tpj71169-bib-0025] Liu, H. , Yang, Y. , Liu, D. , Wang, X. & Zhang, L. (2020) Transcription factor *TabHLH49* positively regulates dehydrin *WZY2* gene expression and enhances drought stress tolerance in wheat. BMC Plant Biology, 20, 259. Available from: 10.1186/s12870-020-02474-5 PMC7275420 32503498

[tpj71169-bib-0026] Liu, X. , Bulley, S.M. , Varkonyi‐Gasic, E. , Zhong, C. & Li, D. (2023) Kiwifruit bZIP transcription factor *AcePosF21* elicits ascorbic acid biosynthesis during cold stress. Plant Physiology, 192, 982–999. Available from: 10.1093/plphys/kiad121 PMC10231468 36823691

[tpj71169-bib-0027] Liu, Y. , Chen, B. , Qin, Z. , Jiang, P. , Yang, Y. , Wang, C. et al. (2025) TaFAR5‐TaFAR3 module regulates cuticular wax biosynthesis and drought tolerance in wheat. New Phytologist, 248, 1802–1821. Available from: 10.1111/nph.70512 40887879

[tpj71169-bib-0029] Livak, K.J. & Schmittgen, T.D. (2001) Analysis of relative gene expression data using real‐time quantitative PCR and the 2(‐Delta Delta C(T)) method. Methods, 25, 402–408. Available from: 10.1006/meth.2001.1262 11846609

[tpj71169-bib-0030] Luan, Y.T. , Yu, H. , An, H.L. , Chen, Z.J. , Zhao, D.Q. & Tao, J. (2025) Phosphorylation of the transcription factor PoWRKY31 by PoKIN10 activates lignin biosynthesis to improve tree peony drought tolerance. New Phytologist, 248, 1784–1801. Available from: 10.1111/nph.70546 40906616

[tpj71169-bib-0031] Luo, P. , Shen, Y. , Jin, S. , Huang, S. , Cheng, X. , Wang, Z. et al. (2016) Overexpression of *Rosa rugosa* anthocyanidin reductase enhances tobacco tolerance to abiotic stress through increased ROS scavenging and modulation of ABA signaling. Plant Science, 245, 35–49. Available from: 10.1016/j.plantsci.2016.01.007 26940490

[tpj71169-bib-0032] Luo, P. , Zhang, H. , Chen, Y. , Cui, Y. & Chen, W. (2024) *RhbHLH92* positively regulates the dehydration tolerance by interacting with RhMYB123 in rose petals (*Rosa hybrida*). Environmental and Experimental Botany, 228, 106049. Available from: 10.1016/J.ENVEXPBOT.2024.106049

[tpj71169-bib-0034] Ning, G. , Xiao, X. , Lv, H. , Li, X. , Zuo, Y. & Bao, M. (2012) Shortening tobacco life cycle accelerates functional gene identification in genomic research. Plant Biology (Stuttgart, Germany), 14, 934–943. Available from: 10.1111/j.1438-8677.2012.00571.x 23107371

[tpj71169-bib-0035] Niu, M.X. , Feng, C.H. , He, F. , Zhang, H. , Bao, Y. , Liu, S.J. et al. (2024) The miR6445‐NAC029 module regulates drought tolerance by regulating the expression of glutathione S‐transferase U23 and reactive oxygen species scavenging in *Populus* . New Phytologist, 242, 2043–2058. Available from: 10.1111/nph.19703 38515251

[tpj71169-bib-0036] Passeri, V. , Martens, S. , Carvalho, E. , Bianchet, C. , Damiani, F. & Paolocci, F. (2017) The R2R3MYB *VvMYBPA1* from grape reprograms the phenylpropanoid pathway in tobacco flowers. Planta, 246, 185–199. Available from: 10.1007/s00425-017-2667-y 28299441

[tpj71169-bib-0037] Peng, X. , Feng, C. , Wang, Y.T. , Zhang, X. , Wang, Y.Y. , Sun, Y.T. et al. (2022) miR164g‐*MsNAC022* acts as a novel module mediating drought response by transcriptional regulation of reactive oxygen species scavenging systems in apple. Horticulture Research, 9, uhac192. Available from: 10.1093/hr/uhac192 PMC9630969 36338839

[tpj71169-bib-0038] Raymond, O. , Gouzy, J. , Just, J. , Badouin, H. , Verdenaud, M. , Lemainque, A. et al. (2018) The *Rosa* genome provides new insights into the domestication of modern roses. Nature Genetics, 50, 772–777. Available from: 10.1038/s41588-018-0110-3 PMC5984618 29713014

[tpj71169-bib-0039] Ren, Y.R. , Yang, Y.Y. , Zhao, Q. , Zhang, T.E. , Wang, C.K. , Hao, Y.J. et al. (2021) *MdCIB1*, an apple bHLH transcription factor, plays a positive regulator in response to drought stress. Environmental and Experimental Botany, 188, 104523. Available from: 10.1016/J.ENVEXPBOT.2021.104523

[tpj71169-bib-0040] Shen, Y. , Sun, T. , Pan, Q. , Anupol, N. , Chen, H. , Shi, J. et al. (2019) *RrMYB5*‐ and *RrMYB10*‐regulated flavonoid biosynthesis plays a pivotal role in feedback loop responding to wounding and oxidation in *Rosa rugosa* . Plant Biotechnology Journal, 17, 2078–2095. Available from: 10.1111/pbi.13123 PMC6790370 30951245

[tpj71169-bib-0042] Song, Q. , Kong, L. , Yang, J. , Lin, M. , Zhang, Y. , Yang, X. et al. (2024) The transcription factor *PtoMYB142* enhances drought tolerance in *Populus tomentosa* by regulating gibberellin catabolism. The Plant Journal, 118, 42–57. Available from: 10.1111/tpj.16588 38112614

[tpj71169-bib-0043] Sun, S. , Qi, X. , Zhang, Z. , Sun, L. , Wang, R. , Li, Y. et al. (2024) A structural variation in the promoter of the leucoanthocyanidin reductase gene AaLAR1 enhances freezing tolerance by modulating proanthocyanidin accumulation in kiwifruit (*Actinidia arguta*). Plant, Cell & Environment, 47, 4048–4066. Available from: 10.1111/pce.15003 38884345

[tpj71169-bib-0044] Sun, X. , Wang, P. , Jia, X. , Huo, L. , Che, R. & Ma, F. (2018) Improvement of drought tolerance by overexpressing *MdATG18a* is mediated by modified antioxidant system and activated autophagy in transgenic apple. Plant Biotechnology Journal, 16, 545–557. Available from: 10.1111/pbi.12794 PMC5787838 28703378

[tpj71169-bib-0045] Tian, J. , Pei, H. , Zhang, S. , Chen, J. , Chen, W. , Yang, R. et al. (2014) TRV‐GFP: a modified tobacco rattle virus vector for efficient and visualizable analysis of gene function. Journal of Experimental Botany, 65, 311–322. Available from: 10.1093/jxb/ert381 PMC3883300 24218330

[tpj71169-bib-0046] Wang, L. , Ran, L. , Hou, Y. , Tian, Q. , Li, C. , Liu, R. et al. (2017) The transcription factor *MYB115* contributes to the regulation of proanthocyanidin biosynthesis and enhances fungal resistance in poplar. New Phytologist, 215, 351–367. Available from: 10.1111/nph.14569 28444797

[tpj71169-bib-0047] Wang, N. , Xu, H. , Jiang, S. , Zhang, Z. , Lu, N. , Qiu, H. et al. (2017) *MYB12* and *MYB22* play essential roles in proanthocyanidin and flavonol synthesis in red‐fleshed apple (*Malus sieversii* f. *niedzwetzkyana*). The Plant Journal, 90, 276–292. Available from: 10.1111/tpj.13487 28107780

[tpj71169-bib-0048] Wang, Z. , Wong, D.C.J. , Wang, Y. , Xu, G. , Ren, C. , Liu, Y. et al. (2021) GRAS‐domain transcription factor *PAT1* regulates jasmonic acid biosynthesis in grape cold stress response. Plant Physiology, 186, 1660–1678. Available from: 10.1093/plphys/kiab142 PMC8260143 33752238

[tpj71169-bib-0050] Xing, W. , Bao, Y. , Luo, P. , Bao, M.Z. & Ning, G.G. (2014) An efficient system to produce transgenic plants via cyclic leave‐originated secondary somatic embryogenesis in *Rosa rugosa* . Acta Physiologiae Plantarum, 36, 2013–2023. Available from: 10.1007/s11738-014-1578-9

[tpj71169-bib-0051] Xu, W. , Dubos, C. & Lepiniec, L. (2015) Transcriptional control of flavonoid biosynthesis by MYB‐bHLH‐WDR complexes. Trends in Plant Science, 20, 176–185. Available from: 10.1016/j.tplants.2014.12.001 25577424

[tpj71169-bib-0052] Yan, Y. , Liu, W. , Wei, Y. & Shi, H. (2020) *MeCIPK23* interacts with whirly transcription factors to activate abscisic acid biosynthesis and regulate drought resistance in cassava. Plant Biotechnology Journal, 18, 1504–1506. Available from: 10.1111/pbi.13321 PMC7292532 31858710

[tpj71169-bib-0053] Yang, A. , Dai, X. & Zhang, W.H. (2012) A R2R3‐type MYB gene, *OsMYB2*, is involved in salt, cold, and dehydration tolerance in rice. Journal of Experimental Botany, 63, 2541–2556. Available from: 10.1093/jxb/err431 PMC3346221 22301384

[tpj71169-bib-0054] Yang, Y. , Chen, M. , Zhu, Q. , Lv, Y. , Liu, C. , Wei, Y. et al. (2025) The transcription factors *AdNAC3* and *AdMYB19* regulate kiwifruit ripening through brassinosteroid and ethylene signaling networks. Plant Physiology, 197, kiaf084. Available from: 10.1093/plphys/kiaf084 PMC11896978 39977254

[tpj71169-bib-0055] Yong, Y. , Bi, H. , Li, M. , Zhu, Y. , Zhou, Q. , Xing, W. et al. (2026) LlR3MYB‐mediated flavonoid biosynthesis confers cold stress tolerance in *Lilium lancifolium* through the LlDREB‐LlCHS2 regulatory cascade. Horticulture Research, 13, uhag065. Available from: 10.1093/hr/uhag065 PMC13249511 42273268

[tpj71169-bib-0056] Yuan, L. , Wang, L. , Han, Z. , Jiang, Y. , Zhao, L. , Liu, H. et al. (2012) Molecular cloning and characterization of *PtrLAR3*, a gene encoding leucoanthocyanidin reductase from *Populus trichocarpa*, and its constitutive expression enhances fungal resistance in transgenic plants. Journal of Experimental Botany, 63, 2513–2524. Available from: 10.1093/jxb/err425 PMC3346219 22268151

[tpj71169-bib-0057] Yuan, X. , Huang, P. , Wang, R. , Li, H. , Lv, X. , Duan, M. et al. (2018) A zinc finger transcriptional repressor confers pleiotropic effects on rice growth and drought tolerance by down‐regulating stress‐responsive genes. Plant & Cell Physiology, 59, 2129–2142. Available from: 10.1093/pcp/pcy133 30020522

[tpj71169-bib-0058] Zhang, H. , Sun, Z. , Feng, S. , Zhang, J. , Zhang, F. , Wang, W. et al. (2022) The C2H2‐type zinc finger protein PhZFP1 regulates cold stress tolerance by modulating galactinol synthesis in *Petunia hybrida* . Journal of Experimental Botany, 73, 6434–6448. Available from: 10.1093/jxb/erac274 35726094

[tpj71169-bib-0059] Zhang, J. , Zhang, H. , Srivastava, A.K. , Pan, Y. , Bai, J. , Fang, J. et al. (2018) Knockdown of rice microRNA166 confers drought resistance by causing leaf rolling and altering stem xylem development. Plant Physiology, 176, 2082–2094. Available from: 10.1104/pp.17.01432 PMC5841683 29367235

[tpj71169-bib-0060] Zhang, L. , Jiang, X. , Liu, Q. , Ahammed, G.J. , Lin, R. , Wang, L. et al. (2020) The *HY5* and *MYB15* transcription factors positively regulate cold tolerance in tomato via the CBF pathway. Plant, Cell & Environment, 43, 2712–2726. Available from: 10.1111/pce.13868 32799321

[tpj71169-bib-0061] Zhang, N. , Zhang, H. , Bai, B. , Li, J. , Cao, X. , Mao, X. et al. (2025) Jasmonic acid activates transcription factor *SlMYB13* to enhance cold resistance in tomato. Plant, Cell & Environment, 48, 7332–7347. Available from: 10.1111/pce.70030 40545701

[tpj71169-bib-0062] Zhang, Y. , Ye, J. , Liu, C. , Xu, Q. , Long, L. & Deng, X. (2020) Citrus PH4‐Noemi regulatory complex is involved in proanthocyanidin biosynthesis via a positive feedback loop. Journal of Experimental Botany, 71, 1306–1321. Available from: 10.1093/jxb/erz506 PMC7031078 31728522

[tpj71169-bib-0063] Zhao, M. , Wang, L. , Feng, M. , Li, X. , Huang, C. , Lian, S. et al. (2025) Trehalose‐6‐phosphate synthase *VvTPS10* regulates cold tolerance by participating in the CBF pathway and *VvPUB19*‐mediated ubiquitination in grapevine. Plant, Cell & Environment, 48, 8086–8102. Available from: 10.1111/pce.70108 40767122

[tpj71169-bib-0064] Zhu, M. , Meng, X. , Cai, J. , Li, G. , Dong, T. & Li, Z. (2018) Basic leucine zipper transcription factor *SlbZIP1* mediates salt and drought stress tolerance in tomato. BMC Plant Biology, 18, 83. Available from: 10.1186/s12870-018-1299-0 PMC5941487 29739325

