# Supplementary material for: The RcDREB1E‐RcMYB28 module improves drought tolerance in Rosa chinensis via RcLAR / RcCORA1 ‐mediated proanthocyanidin biosynthesis and stress responses
Source: Plant J. 2026 Oct 14;128(1):e71169. doi: 10.1111/tpj.71169 (PMC13662611; doi:10.1111/tpj.71169)
Supplement: Supplementary file 1 [file TPJ-128-0-s001.docx]

**Figure S1** qRT-PCR analysis of *RhANR* and *RhLAR* in the rose (*Rosa hybrida*) petals and leaves during the dehydration treatment. **(a-b)** qRT-PCR analysis of *RhANR* and *RhLAR* in the rose (*Rosa hybrida*) petals during the dehydration treatment. **(c-d)** qRT-PCR analysis of *RhANR* and *RhLAR* in the rose (*Rosa hybrida*) leaves during the dehydration treatment. Values are means±SD from 3 independent biological replicates. Statistical significance was determined using a Student’s t-test (* *P* < 0.05, ** *P* < 0.01, *** *P* < 0.001).

**Figure S2** Heatmap analysis of differentially expressed MYB transcription factors under drought stress at 0 d, 30 d, and 60 d.

**(a)**

**(b)**

**Figure S3** Phylogenetic tree constructed based on the differentially expressed MYB transcription factors of ‘Old blush’ under drought stress and MYB proteins from *Arabidopsis thaliana*. **(a)** Phylogenetic tree constructed based on the differentially expressed R2R3-MYB transcription factors of ‘Old blush’ under drought stress and MYB proteins from *Arabidopsis thaliana*. **(b)** Phylogenetic tree constructed based on the differentially expressed 1R-MYB transcription factors of ‘Old blush’ under drought stress and MYB proteins from *Arabidopsis thaliana*.

**Figure S4** Multiple alignments of RcMYB28 and MYBs from other plants.

**Figure S5** Phenotypic observation of *RcMYB28*-overexpressing tobacco leaves

**Figure S6** qRT-PCR analysis of *NtANS* and *NtUFGT* in the *RcMYB28*-overexpressing tobacco lines. **(a-b)** qRT-PCR analysis of *NtANS* and *NtUFGT* in the *RcMYB28*-overexpressing tobacco petals. **(c-d)** qRT-PCR analysis of *NtANS* and *NtUFGT* in the *RcMYB28*-overexpressing tobacco leaves. Values are means±SD from 3 independent biological replicates. Statistical significance was determined using a Student’s t-test (* *P* < 0.05, ** *P* < 0.01).

**Figure S7**  Multiple sequence alignment of RcMYB28 from *Rosa chinensis* and RrMYB28 from *Rosa rugosa*

**Figure S8** qRT-PCR analysis of genes implicated in stress-response in the *RcMYB28-*overexpressing rose lines (*R. rugosa*) after drought. The different letters indicate significant differences (*P* < 0.05) according to Tukey’s test.

**Figure S9** Expression patterns of *RcDREB1E* under dehydration treatment. The different letters indicate significant differences (*P* < 0.05) according to Tukey’s test.

**Figure S10** Multiple alignments of RcDREB1E and DREBs from other plants.

**Figure S11** Phylogenetic tree constructed based on amino acids of RcDREB1E (marked with pentagram) and DREBs of other plants.

**Figure S12** Effects of RcDREB1E on the promoter activity of *RcLAR* and *RcANR* via Dual-LUC assays. **(a)** Luciferase activity analysis using 35S:*RcDREB1E* as the effector and *proRcLAR*:LUC as a reporter. Empty effector vector (62SK) was used as *the control.* **(b)** Luciferase activity analysis using 35S:*RcDREB1E* as the effector and *proRcANR*:LUC as a reporter. Empty effector vector (62SK) was used as the control. The different letters indicate significant differences (*P* < 0.05) according to Tukey’s test.

**Figure S13** qRT-PCR analysis of *RcANR* and *RcLAR* in the TRV*-RcDREB1E* seedlings. Values are means±SD from 3 independent biological replicates. Statistical significance was determined using a Student’s t-test (* *P* < 0.05).

**Table S1 Primer sequences used in this study**

| Primer name |  | Sequence(5’-3’) |
| --- | --- | --- |
| **For isolation of *RcDREB1E*, *RcMYB28*, and *RcCORA1* in *Rosa chinensis*** | | |
| RcMYB28-ORF-F | Forward | ATGGGTAGAGGTCCTCGGT |
| RcMYB28-ORF-R | Reverse | TCAACTCAACATAACATTTAGATCA |
| RcDREB1E-ORF-F | Forward | ATGAACTCCAACAACCAGCTC |
| RcDREB1E-ORF-R | Reverse | TCATATTGAGTAGCTCCACAGGTTG |
| RcCORA1-ORF-F | Forward | CAGGAACTTCACTCAGAAATCA |
| RcCORA1-ORF-R | Reverse | CTTACATCAGGTACGTACGCTT |
| **For vector constructions of subcellular localization** | | |
| RcMYB28-SL-F | Forward | GGTACCATGGGTAGAGGTCCTCGGT |
| RcMYB28-SL-R | Reverse | GGATCCTCAACTCAACATAACATTTA |
| **For vectors construction of transformation in tobacco and rose** | | |
| RcMYB28-OE-F | Forward | GGTACCATGGGTAGAGGTCCTCGGT |
| RcMYB28-OE-R | Reverse | GGATCCTCAACTCAACATAACATTTA |
| RcDREB1E-ORF-F | Forward | GGATCCATGAACTCCAACAACCAGCTC |
| RcDREB1E-ORF-R | Reverse | GTCGACTCATATTGAGTAGCTCCAC |
| RcCORA1-ORF-F | Forward | GGTACCCAGGAACTTCACTCAGAAATCA |
| RcCORA1-ORF-R | Reverse | GTCGACCTTACATCAGGTACGTACGCTT |
| **For vectors construction of VIGS in *Rosa chinensis*** | | |
| RcCORA1-VS-F | Forward | ACTTTTCTTCTTGTCGCCCT |
| RcCORA1-VS-R | Reverse | CTCAGTTTCAGTCTCAACAGCA |
| RcDREB1E-VS-F | Forward | GAATTCCAGGGAGCCCAACAAGAA |
| RcDREB1E-VS-R | Reverse | GGATCCCCGCCATATTAGTCAGCAAC |
| **For vectors construction of transactivation activity in yeast cell** | | |
| RcMYB28-dFL-F | Forward | CATATGGGTAGAGGTCCTCGGT |
| RcMYB28-dFL-R | Reverse | GGATCCTCAACTCAACATAACATTTA |
| RcMYB28-dΔC-F | Forward | CATATGGGTAGAGGTCCTCG |
| RcMYB28-dΔC-R | Reverse | TTGAGCTTGTGCCATTTTCT |
| RcMYB28-dΔN-F | Forward | CATATGAGTTCACCTCAGTCCAA |
| RcMYB28-dΔN-R | Reverse | GGATCCTCAACTCAACATAACATTT |
| **For vectors construction of Y1H in yeast cell** | | |
| RcMYB28-AD-F | Forward | CATATGGGTAGAGGTCCTCGGT |
| RcMYB28-AD-R | Reverse | GGATCCTCAACTCAACATAACATTTA |
| proRcANR-Y1-F | Forward | GAATTCTTCTTTGGAGATCAAGTACCCG |
| proRcANR-Y1-R | Reverse | ACTAGTCTTGTTTGAGACGATGGGTTG |
| proRcLAR-Y1-F | Reverse | GAGCTCAGTTATTGATGCTTGGGCTTTT |
| proRcLAR-Y1-R | Forward | ACTAGTTGGAGGCCCTAAGTTGAAACA |
| proRcCORA1-Y1-F | Reverse | GAATTCCATTCCATTGATTTTGCT |
| proRcCORA1-Y1-R | Forward | ACTAGTAGGGCGACAAGAAGAAAA |
| RcDREB1E-AD-F | Forward | CATATGAACTCCAACAACCAGCT |
| RcDREB1E-AD-R | Reverse | GAATTCTCATATTGAGTAGCTCCACA |
| proRcMYB28-Y1-F | Forward | GAGCTCGCTAAGAGCCTAAAACACA |
| proRcMYB28-Y1-R | Reverse | ACTAGTTTTGTTGAGTCCATCCTTCG |
| **For vectors construction of LUC and GUS in tobacco** | | |
| RcMYB28-SK-F | Forward | GGTACCATGGGTAGAGGTCCTC |
| RcMYB28-SK-R | Reverse | GGATCCTCAACTCAACATAACATTTAG |
| RcDREB1E-SK-F | Forward | ATGAACTCCAACAACCAGCTC |
| RcDREB1E-SK-R | Reverse | TCATATTGAGTAGCTCCACAGG |
| proRcANR-GUS-F | Forward | GAATTCTTCTTTGGAGATCAAGTACCCG |
| proRcANR-GUS-R | Reverse | ACTAGTCTTGTTTGAGACGATGGGTTG |
| proRcLAR--GUS-F | Forward | GGATCCAGTTATTGATGCTTGGGC |
| proRcLAR--GUS-R | Reverse | CCATGGTGGAGGCCCTAAGTTGA |
| proRcMYB28-GUS-F | Forward | GGATCCGCTAAGAGCCTAAAACACA |
| proRcMYB28-GUS-R | Reverse | CCATGGTTTGTTGAGTCCATCCTTCG |
| proRcCORA1-GUS-F | Forward | GGTACCCATTCCATTGATTTTGCTACTTG |
| proRcCORA1-GUS-R | Reverse | CCATGGAGGGCGACAAGAAGAAAAGT |
| proRcANR-LUC-F | Forward | TTCTTTGGAGATCAAGTA |
| proRcANR-LUC-R | Reverse | CTTAAGTTCTTCTCCTTC |
| proRcLAR-LUC-F | Forward | GGATCCAGTTATTGATGCTTGGGC |
| proRcLAR-LUC-R | Reverse | CCATGGTGGAGGCCCTAAGTTGA |
| proRcMYB28-LUC-F | Forward | GGATCCGCTAAGAGCCTAAAACACA |
| proRcMYB28-LUC-R | Reverse | CCATGGTTTGTTGAGTCCATCCTTCG |
| proRcCORA1-LUC-F | Forward | CATTCCATTGATTTTGCTACTTG |
| proRcCORA1-LUC-R | Reverse | AGGGCGACAAGAAGAAAAGT |
| **For qRT-PCR analysis** | | |
| RcMYB28-qF | Forward | ACTTCTGCAAAATACTGGACTGTGA |
| RcMYB28-qR | Reverse | CATTCCCAAGCCAATCCTCAT |
| RcDREB1E-qF | Forward | GCTGCGACGACAACGAGAG |
| RcDREB1E-qR | Reverse | AAGTAATGACGAGGGGGAGACA |
| RcGAPDH-qF | Forward | TGAAGGGTGGTGCCAAGAA |
| RcGAPDH-qR | Reverse | AAGGGGAGCAAGACAGTTGG |
| NtEF1α-qF | Forward | TGGTTGTGACTTTTGGTCCCA |
| NtEF1α-qR | Reverse | ACAAACCCACGCTTGAGATCC |
| NtCHS-qF | Forward | AAGCAAGAGAAACTAAAGGCTACAAG |
| NtCHS-qR | Reverse | AAATCCAAAAAGCACACCCCAT |
| NtCHI-qF | Forward | CGGGTGCCTCCATTCTTTTTACT |
| NtCHI-qR | Reverse | CCTGACACTCTTTCGGCGATACTAC |
| NtF3H-qF | Forward | CCAGACAAACCAGATGGATGGATAG |
| NtF3H-qR | Reverse | CAAGGGTAAGGTCGGGCTGTG |
| NtF3’H-qF | Forward | TGGCTATTTCATTCCAAAAGGCTCA |
| NtF3’H-qR | Reverse | CTTCAAAGTCATTTCCTCGCACATC |
| NtDFR-qF | Forward | GCAGTTGCTTCCCTTTTCTACC |
| NtDFR-qR | Reverse | TTCCCCATTGGTTGACTTTCC |
| NtANR-qF | Forward | CATTTGACTTTCCCAAACGC |
| NtANR-qR | Reverse | ATTGGGCTTTTGAGTTGTGC |
| NtLAR-qF | Forward | TCAAGGTCCTTTACGCCATC |
| NtLAR-qR | Reverse | ACGAACCTGCTTCTCTTTGG |
| RhANR-qF | Forward | AATGGGTTGGTCTGATGTTGAGT |
| RhANR-qR | Reverse | ATTTCCTGTAATTAAAGATGTGGCG |
| RhLAR-qF | Forward | AAGGTGAGGCGTGCGATTGAGAA |
| RhLAR-qR | Reverse | CGGAACAACCTCTGAAGGATGCT |
| RrCORA1-qF | Forward | TCGCCTTCGCTGTTCTCCTC |
| RrCORA1-qR | Reverse | CCTCCATGACCTCCGTGTCC |
| RrDHAR-qF | Forward | TCACCCATAGGGTTCTTCTCAC |
| RrDHAR-qR | Reverse | AATCCCAGCAAGCACATCAG |
| RrLEA2-qF | Forward | TGAGGCAGAGGAAAAAGGGAAG |
| RrLEA2-qR | Reverse | GTCTTTTTGATCTTCTCCGACTGCT |
| RrMDHAR-qF | Forward | GGAGCTGATGCAAAGAACAT |
| RrMDHAR-qR | Reverse | CCTCCTCCAACAACTACTACCT |
| RrP5CS-qF | Forward | TGGGAAGAGGGGGTATGACTGC |
| RrP5CS-qR | Reverse | GCTTCAATGGCATCGGCTATAT |
| RrPIP2;1-qF | Forward | TGGCATGATCTTTGTCCTTGTT |
| RrPIP2;1-qR | Reverse | GCTCTGGGTAATGACACCTTCCT |

**Table S2 Information about the *RcMYB28* promoter-interacting proteins**

| No. | Accession no. | Details | Clones |
| --- | --- | --- | --- |
| 1 | XM_024312583.1 | zinc finger protein ZAT10-like | 3 |
| 2 | XM_024341820.2 | WRKY transcription factor 75 | 1 |
| 3 | XM_024317263.1 | dehydration-responsive element-binding protein 1E-like | 6 |
| 4 | XM_024324014.2 | WRKY transcription factor 51 | 1 |
| 5 | XM_024335704.1 | calcium-dependent protein kinase 8-like | 1 |
